# Safety, Efficacy and Immunogenicity of a *Salmonella* Paratyphi A vaccine

**DOI:** 10.1056/NEJMoa2502992

**Published:** 2025-10-30

**Authors:** Naina McCann, Margarete Paganotti Vicentine, Narges Ebrahimi, Melanie Greenland, Brian Angus, Andrea M Collins, Thomas Darton, Katherine Emary, Saul N Faust, Amy Flaxman, Noshi Maria, Christopher A Green, Claudia Juarez Molina, Ravindra Paidisetti, Rajeka Lazarus, Grace C Macaulay, Florence Mclean, V. Krishna Mohan, M. Gangadhara Naidu, Maheshi N Ramasamy, D. Yogeswara Rao, Nisha Singh, Sophie Vernon, Young Chan Kim, Myron M Levine, Xinxue Liu, Andrew J Pollard, VASP Study Team

**Affiliations:** 1 Oxford Vaccine Group, Department of Paediatrics, Centre for Clinical Vaccinology and Tropical Medicine, Churchill Hospital, Headington, Oxford, UK OX3 7LE; 2 NIHR Oxford Biomedical Research Centre, Oxford, UK; 3 Liverpool University Hospitals Foundation Trust; 4 Liverpool School of Tropical Medicine, Liverpool, UK; 5 Sheffield Teaching Hospitals NHS Foundation Trust, Sheffield, UK; 6 NIHR Southampton Clinical Research Facility and Biomedical Research Centre, University Hospital Southampton NHS Foundation Trust; 7 Faculty of Medicine and Institute for Life Sciences, University of Southampton, Southampton, UK; 8 NIHR/Wellcome Clinical Research Facility, University Hospitals Birmingham NHS Foundation Trust, Birmingham, UK; 9 Bharat Biotech International Ltd, Genome Valley, Hyderabad, India; 10 Bristol Medical School, University of Bristol; 11 UKHSA Southwest Regional Laboratory and Vaccine Development and Evaluation Centre, Bristol, UK; 12 Oxford University Hospitals NHS Foundation Trust, Oxford, UK; 13 Center for Vaccine Development and Global Health, University of Maryland School of Medicine, Baltimore, MD, USA

## Abstract

**Background:**

*Salmonella* Paratyphi A (SPA) is responsible for over 2 million cases of enteric fever annually. There are no licensed vaccines against SPA.

**Methods:**

We conducted a double-blind randomised clinical trial to evaluate the efficacy, safety and immunogenicity of a two-dose regimen of an SPA oral live attenuated vaccine (CVD 1902) using a controlled human infection model (CHIM). Healthy UK adult participants were randomised 1:1 to receive two doses of vaccine or placebo 14 days apart. Twenty-eight days after second dose, all participants received oral challenge with SPA. The primary endpoint was the proportion of participants diagnosed with paratyphoid within 14 days post-challenge. Secondary endpoints included vaccine safety, tolerability and immunogenicity.

**Results:**

Among 72 randomised participants, 70 (34 CVD 1902 and 36 placebo) were challenged. Median age of the participants was 31.5 years (range 20-54 years) and 46% were female. There were similar numbers of adverse events in the two groups and no vaccine related serious adverse events were identified. CVD 1902 induced serum IgG and IgA responses to the O-antigen of SPA. There was no associated rise in either antibody titre in the placebo group. In the challenge model, the attack rate was 20.6% and 75% in the CVD 1902 and placebo arms, respectively, providing a vaccine efficacy of 72.5% (95% CI 45.5 - 86.2%, p <0.001) in the intention-to-treat and 69.2% (95% CI 41.7-83.8) in the per protocol analysis.

**Conclusions:**

A two-dose regimen of CVD 1902 conferred protection against paratyphoid infection in an UK adult CHIM without evident safety concerns.(Supported by NIHR Oxford Biomedical Research Council, and others; Trial registration no: ISRCTN Registry 15485902)

Enteric fever, a systemic febrile illness caused by serovars *Salmonella enterica* Typhi and Paratyphi A, B and C, is a major public health concern, causing over 100,000 deaths and over 8 million disability-adjusted life years (DALYs) annually (1). Ultimately control of this disease requires access to clean water for all, but over two billion people remain without access to safely managed water (2). Vaccination offers an alternative control mechanism and the recent development and implementation of typhoid conjugate vaccines (TCVs) is a considerable step towards this (3). However, up to 30% of enteric fever disease and over two million cases a year are caused by *Salmonella enterica* Paratyphi A (SPA), for which there is currently no licensed vaccine (1).

There are several vaccines against SPA in development (4). One vaccine candidate is CVD 1902, an engineered oral live attenuated vaccine developed by the University of Maryland. This vaccine, constructed from the wild-type reference SPA strain ATCC 9150 by deletion of the guaBA chromosomal operon and clpX gene, has been shown to be safe and immunogenic in a phase 1 trial (5). Evidence from a prior re-challenge trial conducted in the UK shows that prior exposure to wild-type SPA protects from infection following re-challenge, providing further rationale for a live attenuated vaccine (6). Oral vaccines also offer logistical advantages such as ease of administration and simpler manufacturing processes than subunit vaccines (7).

Efficacy testing of vaccine candidates against SPA is problematic due to the relatively low incidence of paratyphoid fever and thus the large number of participants who would be needed for field trials (8, 9). The WHO recently endorsed the use of vaccine efficacy data from controlled human infection models (CHIMs) to support licensure of SPA vaccines, when combined with field safety, immunogenicity data and post-licensure effectiveness studies.

In this study we used a CHIM to evaluate whether CVD 1902 is efficacious at preventing SPA infection and illness in healthy adult volunteers in the UK. In addition, we assessed the safety and immunogenicity of CVD 1902.

## Methods

### Study Design and Oversight

This was an observer and participant blinded, randomised, controlled phase 2b trial carried out at six research centres across the UK (Oxford, Birmingham, Southampton, Bristol, Sheffield and Liverpool).

Written informed consent was obtained from all participants, and the trial was conducted in accordance with the principles of the Declaration of Helsinki. The study protocol was approved by the sponsor (University of Oxford), the Berkshire Ethics Committee (21/SC/0330) and the Medicines and Healthcare Products Regulatory Agency (EudraCT 2021-003259-41). The clinical trial was registered with the ISRCTN Registry (15485902). An independent data and safety monitoring committee provided safety oversight of the trial and had access to unblinded data throughout (see Supplementary Appendix). Full details can be found in the published protocol paper (10) and the protocol at nejm.org. The authors vouch for the integrity and accuracy of the data presented and for the fidelity of the trial to the protocol.

### Participants

Healthy adult volunteers between the ages of 18 and 55 years with no prior history of enteric fever were recruited. Volunteers were asked to complete an online questionnaire, interviewed by telephone to confirm their medical history, and invited for an in-person visit, where extensive eligibility evaluation was undertaken. A full list of inclusion and exclusion criteria can be found in the Supplementary Appendix. Participants were recruited and followed up at their local site but all participants received vaccination and challenge in Oxford.

### Randomisation and masking

On the day of enrolment participants were randomised 1:1 to receive either vaccination with CVD 1902 or placebo. A randomisation list using varying block sizes (2 and 4) was used.

Preparation of the vaccine was carried out by an unblinded laboratory team. Randomisation and vaccination of participants were carried out by an unblinded clinical team who were not involved in outcome evaluation. Unblinding was scheduled when the last participant had completed their day 28 post-challenge visit.

### Procedures

The study vaccine was given as a two-dose schedule, and the second dose of CVD 1902 or placebo was given 14 days after the first dose. Oral vaccination with CVD 1902 was administered to participants as a 30mL solution (minimum dose of 2 × 10^10^ colony forming units). Placebo was a 1.3% wt/vol sodium bicarbonate solution of 30mL. One minute prior to each vaccination/placebo participants ingested 120mL oral sodium bicarbonate solution to neutralise gastric acid. Participants were monitored for 30 minutes following vaccination and completed an e-diary of their symptoms for 7 days following each vaccination (Day 0 to Day 6) (see Supplementary Appendix).

At 4 weeks (23-60 days) following the second vaccination, all participants were challenged orally with 1-5 × 10^3^ CFU of *S*. Paratyphi A strain NVGH308 (a wild-type strain isolated from a patient with acute paratyphoid fever in Nepal in 2006 and labelled ED199, manufactured to GMP standard by GeniBet BioPharmaceuticals, Portugal). One minute prior to ingestion of the challenge inoculum, participants drank 120mL of sodium bicarbonate. Participants were monitored for 15 minutes following challenge and asked to complete an e-diary of symptoms including twice-daily temperatures for 21 days following challenge (see Supplementary Appendix).

Participants were managed in the outpatient setting and reviewed in person daily at recruiting sites for the 14 days following challenge for clinical assessment and a daily blood culture. Paratyphoid fever was diagnosed if any of the pre-specified criteria were met; a positive blood culture for SPA collected more than 72 hours post-challenge or a fever of 38°C or higher persisting for 12 hours or longer.

Participants diagnosed with SPA were given a 7-14-day course of ciprofloxacin as first-line treatment (the treatment course was extended from 7 days to 14 days during the study – see Supplementary Appendix). For those with a positive blood culture, treatment was commenced at the time that Gram-negative bacilli were identified in the blood culture with confirmatory testing performed subsequently. For those diagnosed clinically, treatment was commenced at the time of the second temperature ≥ 38°C. Those not diagnosed were treated with the same antibiotic course at day 14 post-challenge. All participants provided three separate stool samples (at least 48 hours apart) to prove stool clearance at least one week after completing antibiotics, and attended further follow-up appointments at day 28, 90, 180 and 365 post-challenge.

### Outcomes

The primary outcome was the proportion of participants diagnosed with paratyphoid infection as per the pre-defined composite criteria in the 14 days following oral challenge with wild-type SPA.

Secondary safety outcomes included the safety and tolerability of CVD 1902 based on self-reported solicited symptoms via e-diaries, adverse events within 90 days, and serious adverse events until day 365 post-challenge.

Secondary immunogenicity outcomes included serum IgG and IgA antibody responses to SPA (O-antigen and FliC (flagellin)) following vaccination at day 14 and 42 by an in-house standardised indirect ELISA. Multiple microbiological and clinical secondary outcomes were also evaluated (see Supplementary Appendix). Of note, during the immunogenicity analyses, we identified labelling errors for two participants’ baseline samples. A sensitivity analysis was also carried out after removing these two participants.

### Statistical analysis

Based on findings from the previous SPA challenge studies using the same model and strain, the expected attack rate in the control group was ~58% (6, 11). To demonstrate a protective effect of 70%, 33 participants were needed per group to achieve 90% power (1- β) at a two-sided 5% significance level (α).

The attack rate in each group (CVD 1902 and placebo) was calculated, and the difference in attack rates was compared using Pearson’s chi-squared test. Vaccine efficacy was calculated as the percentage reduction in attack rate within 14 days in the CVD 1902 group compared with the control group, analysed separately in the intention-to-treat (ITT) and per-protocol (PP) populations. For the secondary endpoints, 95% confidence intervals (CIs) were presented without adjusting for multiplicity, and the intervals may not be used in the place of hypothesis testing. Antibody titre comparisons between arms were reported by the geometric mean ratio and 95% CIs. Logistic regression was performed to analyse the association between antibody levels on day of challenge and risk of infection. Data were analysed using R version 4.3.2.

## Results

### Participants

**Figure 1:**
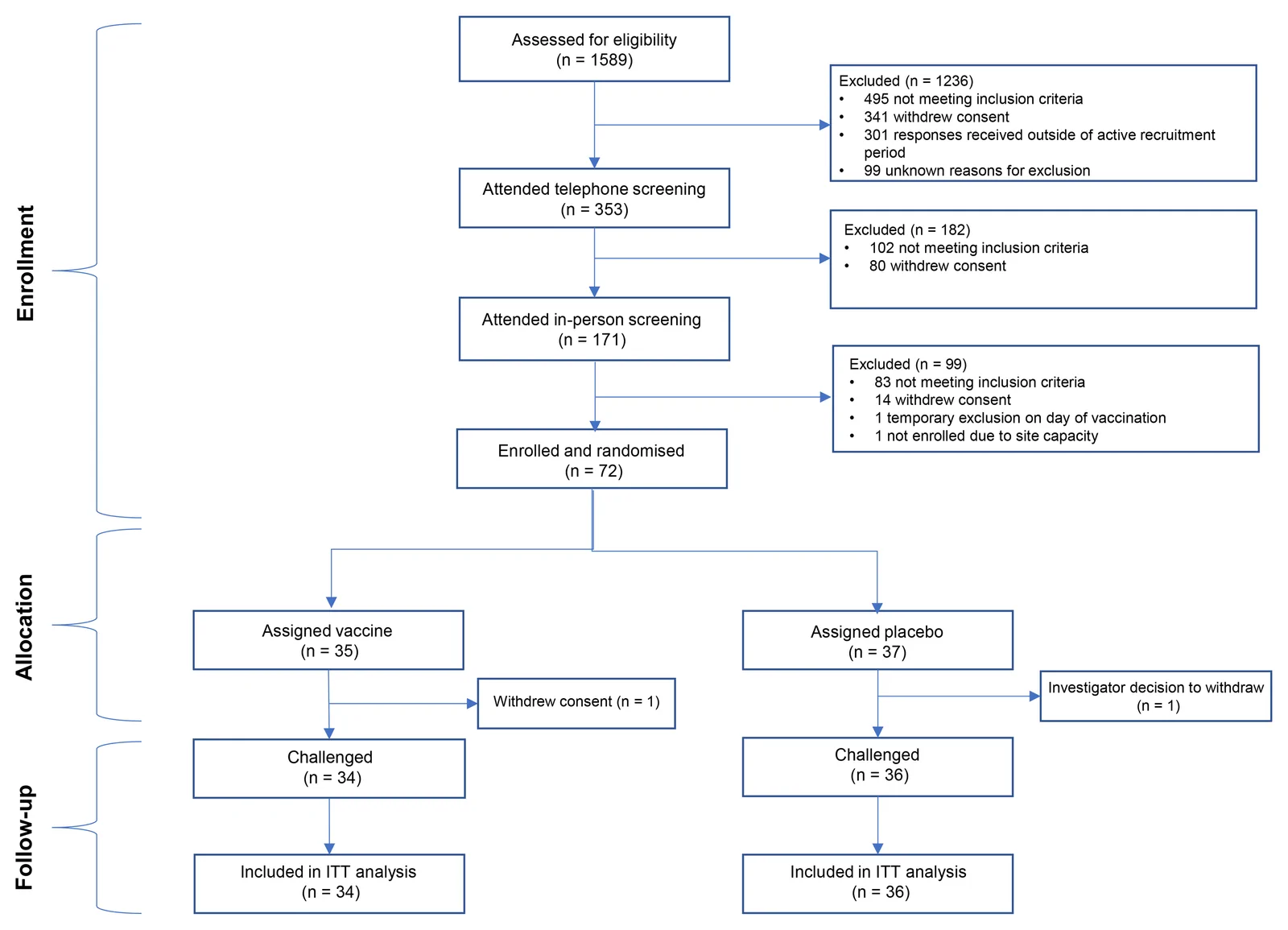
Consort diagram of trial participants.

Between April 2022 and November 2023, 1589 volunteers were assessed for eligibility. Of the 171 participants who were screened in-person, 72 were enrolled, randomised and received at least one dose of CVD 1902 or placebo, including 35 and 37 participants in the CVD 1902 and placebo arm, respectively. Two participants withdrew after the first vaccination dose and 70 participants were included in the per-protocol analysis (Figure 1). Baseline characteristics were similar between the two groups (Table 1).

### Vaccine safety

**Figure 2:**
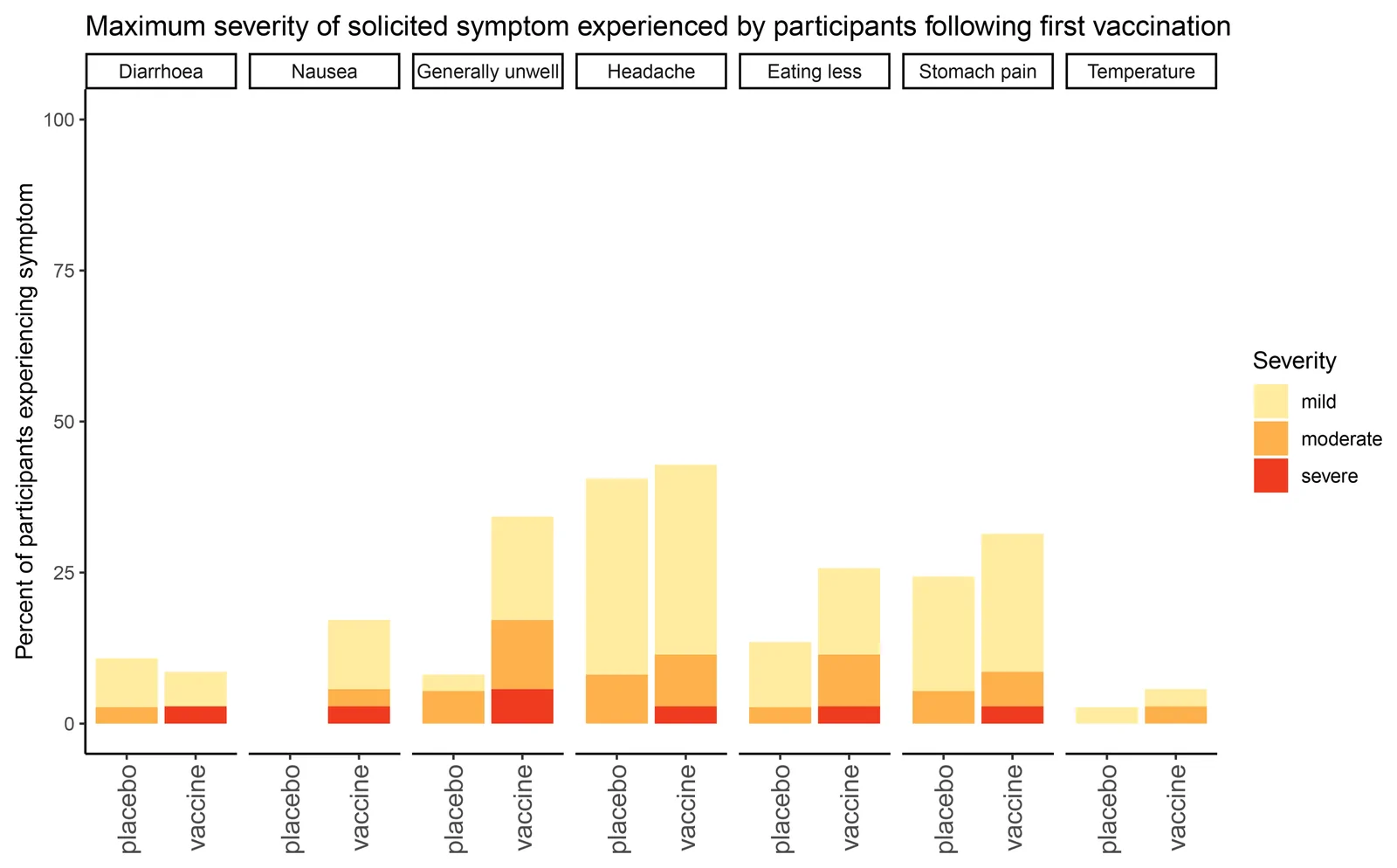
Maximum severity of solicited symptoms following vaccination. Severity of solicited adverse reactions in days 0-6 following A) first vaccine dose and B) second vaccine dose with CVD 1902 as self-reported in participant electronic diaries in the safety analysis population. Severity is presented as the participant’s highest severity across the 7 days for each solicited adverse event. The exact definition of each solicited symptom mild, moderate and severe grading scales are listed in the supplementary appendix.

After the first dose, nausea/vomiting and feeling generally unwell were reported more commonly by the vaccine group than the placebo group but the majority of solicited symptoms were self-rated as mild to moderate severity (Figure 2 and Table S1). Laboratory biochemistry/haematology markers at 7 days post-vaccine were also similar between the two groups (Tables S2 and S3).

Four serious adverse events have been reported in the study (2 in placebo participants and 2 in vaccine participants), none of which were assessed by the investigators as related to vaccination (Table S4). Unsolicited adverse events were similar between the two groups (Table S5).

### Vaccine immunogenicity

**Figure 3:**
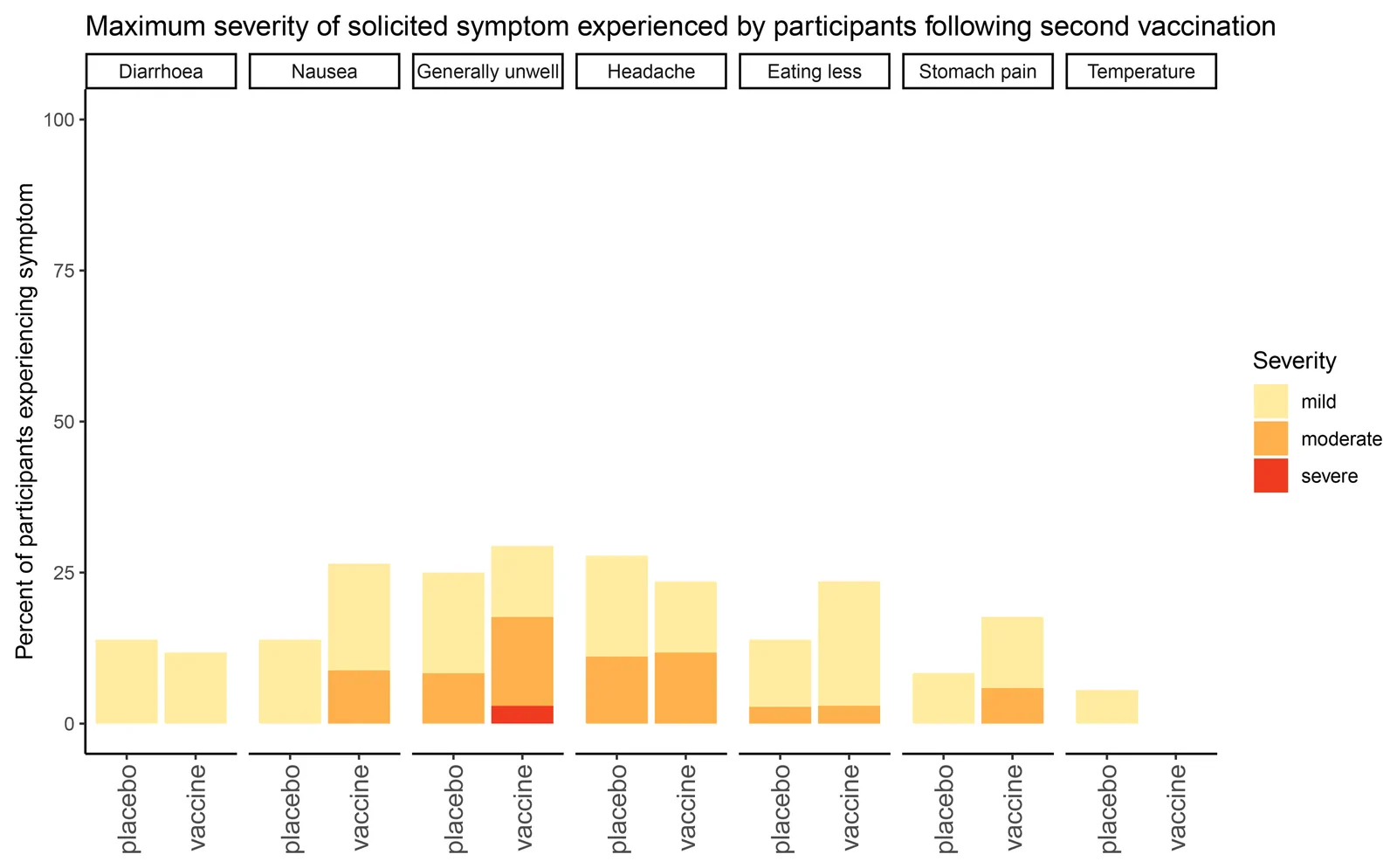
Anti-O IgG (A) and IgA (B) antibody response following vaccination. Anti-SPA specific O-antigen titres measured following at baseline, 14 days post-first vaccine and 42 days post second vaccine (28 days post second vaccine). Individual dots represent individual participant values and error bars represent the mean geometric mean titres and 95% confidence interval for each timepoint. Numbers by error bars are the mean GMT for each timepoint. Plot A) represents anti-O IgG responses and plot B) represents anti-O IgA response.

In the vaccine group there was a rise in both serum IgG and IgA antibodies to the O-antigen of *S*. Paratyphi A post first vaccine (Figure 3 and Table S7). IgG titres in vaccinees rose from a geometric mean titre (GMT) of 126 Elisa units (EU)/mL (95% CI 66 – 241) at baseline to 314 EU/mL (95% CI 167– 590) at day 14 post-vaccination whilst IgA rose from a baseline GMT of 20 (95% CI 16 – 26) to 70 EU/mL (95% CI 49 – 99) at day 14 post first vaccination. At 42 days post-first vaccine (28 days post-second vaccine) anti-O IgG titres remained elevated at 292 EU/mL (95% CI 158-539) and anti-O IgA titres had dropped to 33 EU/mL (24–45) although remained elevated from baseline. There was no rise in anti-O IgG or IgA antibody titres from baseline at either of the post-vaccine timepoints for the placebo group. There was no antibody (either IgG or IgA) response to *S*. Paratyphi A FliC antigen following vaccination (Figure S4 and Table S7). The sensitivity analysis removing the two participants with labelling errors showed similar results (Table S8).

No difference in serum antibody titres (anti-O or anti-FliC IgG or IgA) measured at day 42 (immediately pre-challenge) was detected between the participants who went on to develop infection (susceptible) and those who were protected from infection (protected) (Figure S5). There was no clear association between anti-O IgG and the risk of paratyphoid infection, but participants with higher anti-O IgA were at a lower risk of infection (see Figure S6).

### Vaccine efficacy

**Figure 4:**
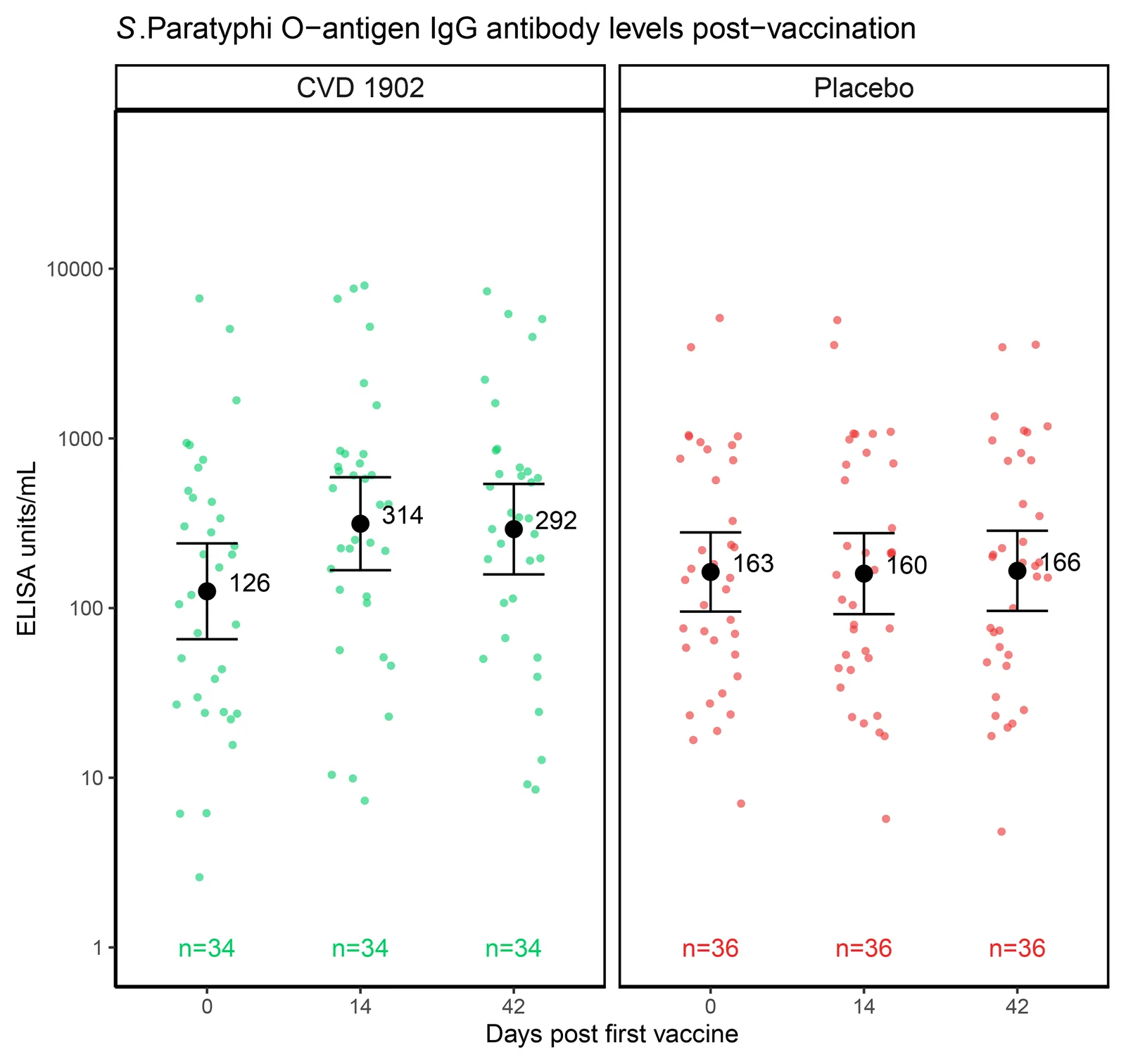
Kaplan Meier curve of time to diagnosis (either via positive blood culture or first fever of ≥ 38°C). Cumulative proportion of participants diagnosed with paratyphoid infection (as per predefined microbiological or clinical criteria) following challenge with *S*. Paratyphi A per group. P value calculated using log-rank test. Shaded areas represent 95% confidence intervals, calculated by the log-transformation method.

Using the composite primary endpoint, paratyphoid infection was diagnosed in 7 (20.6%) in the CVD 1902 group and 27 (75%) in the control group (p <0.001). The calculated vaccine efficacy was 72.5% (95% CI 45.5 -86.2%) in the ITT analysis and 69.2% (95% CI 41.7-83.8) in the PP analysis (Figure 4, Table S9).

Median time from second vaccine dose to challenge was 28 days (range 26-85 days). Sixty-eight participants received challenge within the stated window (23 to 60 days post second-vaccine) and two participants received challenge at 85 days post second-vaccine. Vaccine efficacy was 72% (95% CI 44-86%) after excluding these two participants.

Subgroup analyses were performed by baseline anti-O IgG and interval between second vaccination and challenge, and similar vaccine efficacy was observed across subgroups (Table S10).

Almost all participants reaching the primary disease endpoint (32/34, 94%) were diagnosed via a positive blood culture 72 hours after challenge. Two participants (both in placebo group) were initially diagnosed because of a persistent fever but were later found to also have a positive blood culture.

### Clinical and microbiological endpoints

Clinical manifestations of paratyphoid were similar among diagnosed participants across both groups. Rates of fever associated with paratyphoid diagnosis (≥ 38°C) were 28.5% in CVD 1902 group and 19.5% in placebo group and no participants in either group were diagnosed with severe enteric fever as per pre-specified criteria (Figure S1 and Table S6).

Median time to diagnosis was 7.04 days for CVD 1902 group and 6.08 days for the placebo group (p = 0.33). Median blood culture duration (i.e., time from first positive blood culture to first persistent negative blood culture) was also similar in both groups; 3.01 days in CVD 1902 group and 2.68 days in placebo group (Figure S2). The number of overall participants with a positive stool culture > 72 hours after challenge was lower in the CVD 1902 group (8/34, 23.5%) than the placebo group (18/36, 50%) (Figures S2 and S3).

Following the acute challenge period, three placebo participants relapsed (defined as recurrence of SPA bacteraemia following prior successful treatment) and 7 participants (4 placebo and 3 vaccine) had a positive stool culture for SPA at least 1 week after completing antibiotics. (Table S6). All these participants were re-treated with antibiotics and completed three negative clearance stools by the end of the study.

## Discussion

This randomised controlled trial evaluated the efficacy of a candidate SPA vaccine using a controlled human infection model. In this study, CVD 1902 reduced the risk of infection by 72.5% (95% CI 45.5 -86.2%) compared with placebo.

There are currently no licensed vaccines against *S*. Paratyphi A. The antigens thought most likely to induce a protective immune response are the O:2 antigen of the lipopolysaccharide and the flagellin, as these have been shown to correlate with protection for other enteric pathogens (12). In this study we showed that CVD 1902 induced a serological antibody response (IgG and IgA) to the O-antigen of SPA but pre-challenge levels of these antibodies did not correlate with protection from disease, although there was a trend towards higher anti-O antibodies correlating with protection, particularly for IgA. For intramuscular vaccines against *S*. Typhi, IgA and IgG responses against the capsular polysaccharide Vi-antigen have been shown to correlate with protection from infection (13, 14), but correlates for the live attenuated *S*. Typhi vaccines have been more difficult to elucidate (15). A prior human challenge study investigated serum anti-LPS responses to two *S*. Typhi live attenuated oral vaccines, Ty21a and M01ZH09, and found these did not correlate with protection from infection (16). Protection from a live attenuated vaccine against typhoidal *Salmonella* might be driven by a multi-antigen immune response, and potentially not by serum antibodies alone. Indeed, oral vaccines against *S*. Paratyphi A and *S*. Typhi have previously been shown to elicit strong cell-mediated immune responses (5, 17).

Locally produced mucosal antibodies in the form of secretory IgA are likely to play an important role in protection from SPA, as they do for other enteric pathogens such as cholera as well as respiratory viruses (18, 19). In this study we noted that infected participants had similar clinical and microbiological outcomes between arms. This suggests the mechanism of protection of CVD 1902 may be at the mucosal level. Secretory IgA may be a mediator of this, working to directly block pathogen attachment to epithelial cells or via immune exclusion and agglutination as has been shown to occur with *Salmonella* Typhimurium *in vitro* (20). Future work to illuminate the mucosal response following vaccination, as well as further humoral and cellular aspects of immunity is required to establish a correlate of protection against SPA, which would accelerate vaccine development, evaluation and potentially licensure.

The level of vaccine efficacy we observed with CVD 1902 in this study is higher than we observed previously for a licensed oral typhoid vaccine (Ty21a), and two licensed intra-muscular typhoid vaccines (Vi-TT and Vi-PS) when evaluated in the *S*. Typhi CHIM; Ty21a demonstrated a vaccine efficacy of 35% (95% CI -5 to 60%), Vi-TT of 54.6% (95% CI 26.8 to 71.8%) and Vi-PS of 52.0% (95% CI 23.2 – 70.0%) (13, 16). All these vaccines have shown higher vaccine efficacy in phase 3 studies performed in endemic regions (21). However, the *S*. Paratyphi A CHIM is a different model with a lower challenge dose and participants are less symptomatic than those in the *S*. Typhi model with lower rates of fever, despite having similar levels of bacteraemia (11). One hypothesis for this is that SPA causes a substantial burden of asymptomatic/pauci-symptomatic cases when compared with *S*. Typhi, which, although not requiring hospitalisation, drive ongoing transmission via asymptomatic stool shedding. In this study we found that vaccination may reduce stool shedding in those exposed to the pathogen, potentially interrupting or reducing this transmission cycle.

The limitations of this study include the artificial nature of a human challenge study, and the use of a healthy UK adult population compared with the vaccine target population of school-aged children in endemic areas (see Table S11). Future field studies should be conducted to address this. A CHIM in an endemic setting would also be a useful intermediate step to evaluate how pre-existing immunity affects vaccine response, and this is currently in development. However, there is consistent evidence from other CHIM models used in vaccine development, of alignment of CHIM efficacy with field vaccine efficacy despite these limitations, for example the typhoid Vi-conjugate vaccine (as discussed above), R21/MM (malaria) and the killed whole-cell monovalent cholera toxin B subunit vaccine (22–27).

A bivalent SPA – *S*. Typhi vaccine is the most likely path to licensure for an SPA vaccine, offering a comprehensive solution to enteric fever prevention through a single vaccination, Currently, CVD 1902 requires same-day preparation and therefore further formulation into a product suitable for clinic use is required to proceed towards licensing. This could include combination with an oral live attenuated typhoid vaccine. Intramuscular bivalent conjugates, which combine an SPA O:2 conjugate with a typhoid conjugate vaccine are an alternative option. Two bivalent conjugate vaccine candidates have undergone phase 1 testing but neither of these has undergone efficacy assessment, although studies are planned (28, 29).

CVD 1902 shows efficacy against SPA in the human challenge model. These findings are a step forward towards a much-needed SPA vaccine.

Disclosure forms provided by the authors are available with the full text of this article at NEJM.org.

### Support for Study

This work was supported by the NIHR Oxford Biomedical Research Council and by a grant from the Medical Research Council (MR/RO25347/1). The authors also acknowledge the support of the Oxford University Hospitals NHS Trust who provided laboratory support to the study and are grateful for the involvement and contribution of the study volunteers. We are grateful to the members of the Data Safety Monitoring Committee who provided close monitoring of the study throughout.

## Supplementary Material

Supplement

## Figures and Tables

**Figure:**
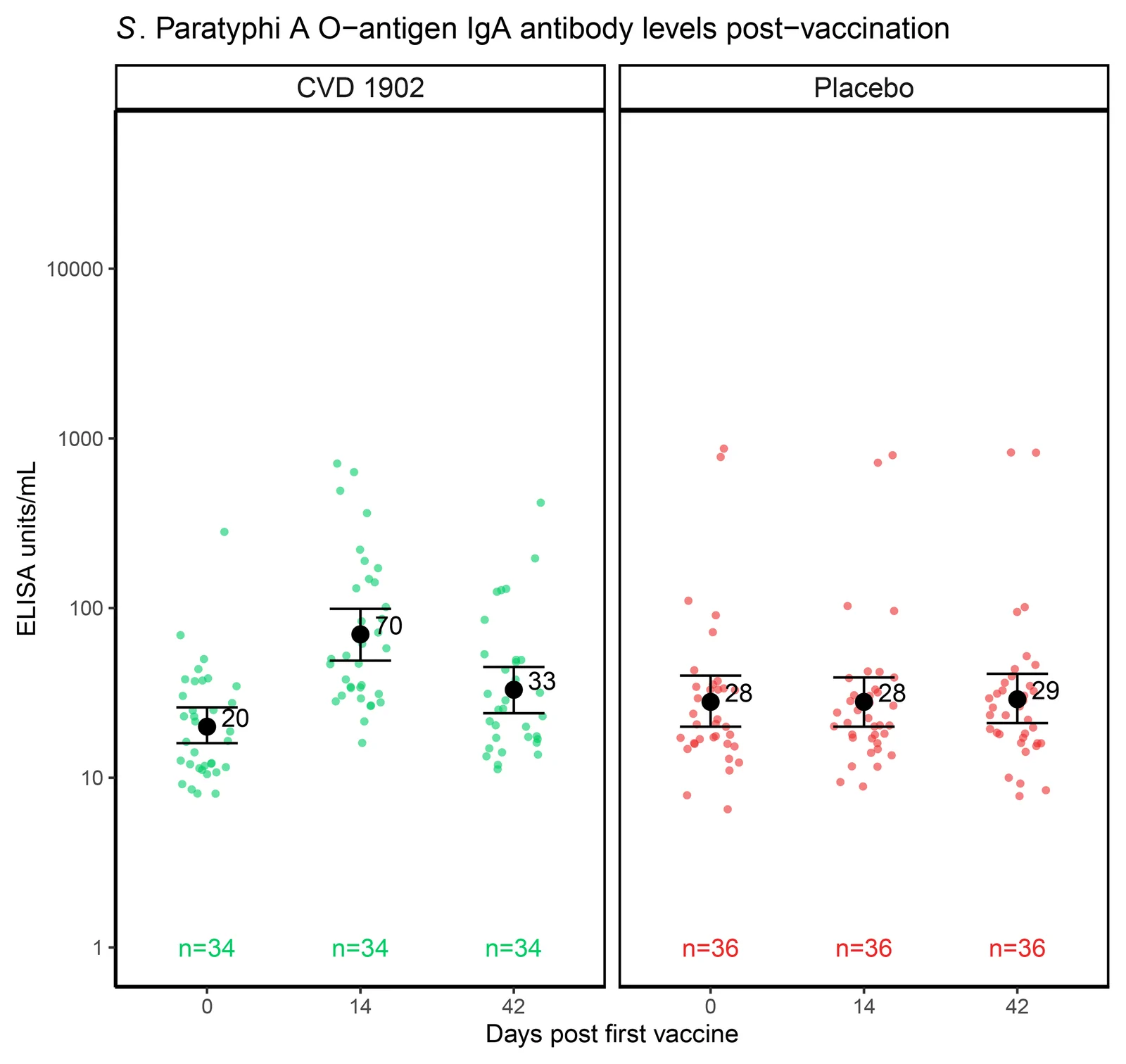


**Figure:**
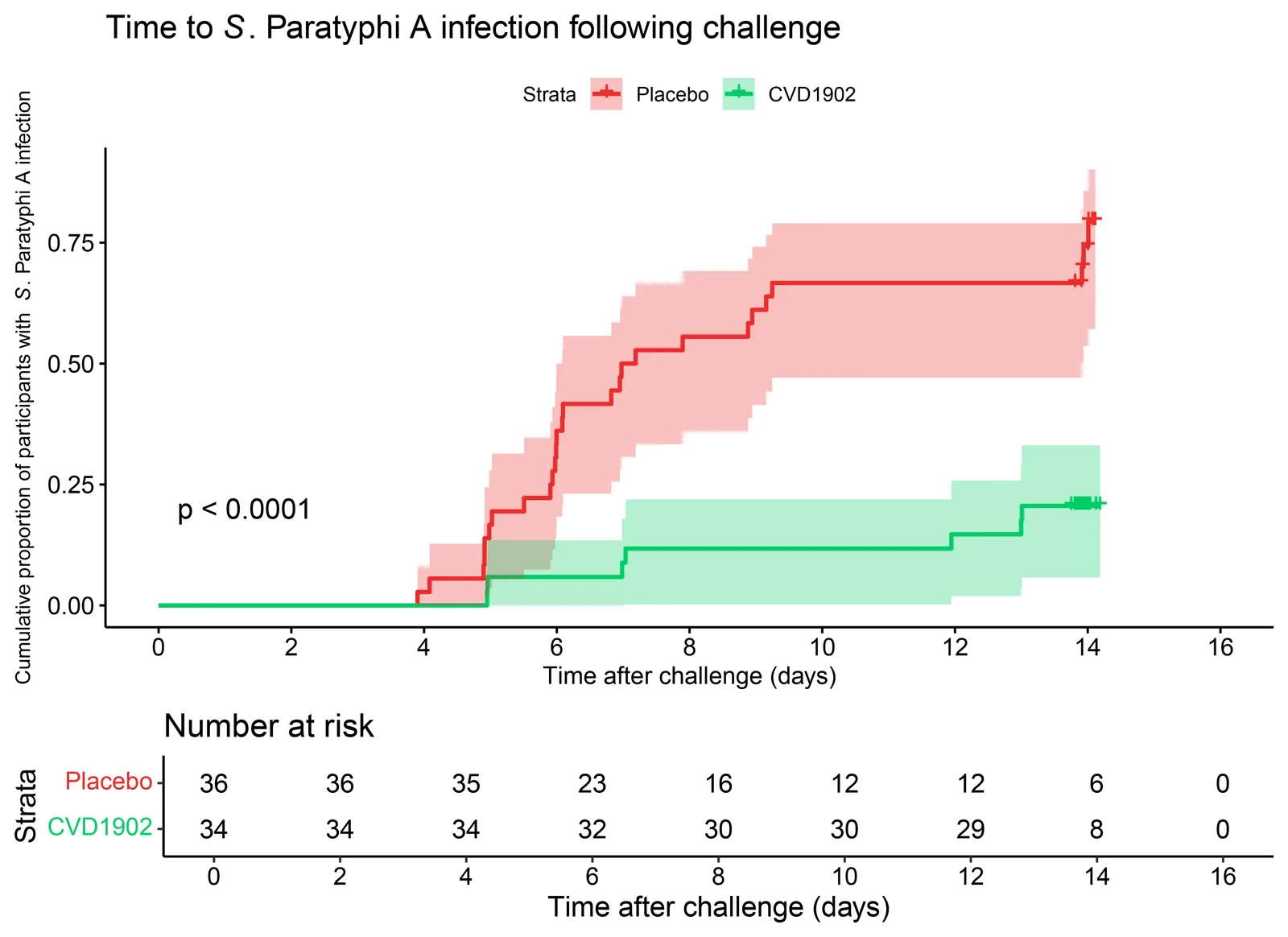


**Table 1:** Baseline participant characteristics

Characteristic	CVD 1902 group (n= 35)	Placebo group (n = 37)	Total (n= 72)
**Age (years)**			
Median (range)	28 (20-54)	33 (20-53)	31.5 (20-54)
**BMI**			
Median (range)	24.5 (19.1-33.2)	26.6 (20.5 - 38.4)	25.4 (19.1-38.4)
**Sex at birth**			
Female	15 (43%)	18 (49%)	33 (46%)
Male	20 (57%)	19 (51%)	39 (54%)
**Ethnic origin**			
White British	25 (71%)	26 (70%)	51 (71%)
White other	2 (6%)	7 (19%)	9 (13%)
Asian	2 (6%)	1 (3%)	3 (4%)
Mixed	4 (11%)	2 (5%)	6 (8%)
Other	2 (6%)	1 (3%)	3 (4%)
**Site enrolled**			
Oxford	25 (71%)	24 (65%)	49 (68%)
Liverpool	4 (11%)	4 (11%)	8 (11%)
Birmingham	3 (9%)	3 (8%)	6 (8%)
Southampton	1 (3%)	3 (8%)	4 (6%)
Bristol	1 (3%)	1 (3%)	2 (3%)
Sheffield	1 (3%)	2 (5%)	3 (4%)

Data are presented as n (%) or median (range) where specified.
