# Supplementary material for: Safety, Efficacy and Immunogenicity of a Salmonella Paratyphi A vaccine
Source: N Engl J Med. Author manuscript; Available in PMC 2026 Nov 5. (PMC7619704; doi:10.1056/NEJMoa2502992)
Supplement: Supplementary file 1 [file EMS209648-supplement-Supplement.pdf]

Supplementary Appendix

Contents

Supplementary Appendix 1
VASP Study Team 2
List of investigators 2
DSMC members 3
Supplementary methods 3
Inclusion Criteria 3
Exclusion Criteria 4
Temporary Exclusion at Vaccination 6
Temporary Exclusion at Challenge 7
Secondary outcomes for the study 7
Enzyme-linked immunosorbent assay (ELISA) for *S.* Paratyphi A antigens 9
Vaccination e-diaries 9
Challenge e-diaries 10
Antibiotic treatment 12
Definition of severe paratyphoid fever 12
Supplementary figures 13
Figure S1. Symptoms of diagnosed participants during challenge 13
Figure S2. Microbiological outcomes during challenge. 15
Figure S3. Stool culture positivity during challenge between groups 16
Figure S4: Anti-FliC IgG (A) and IgA (B) antibody response following vaccination 17
Figure S5. Antibody titres at D42 between protected and susceptible groups 18
Figure S6. Logistic regression modelling of relationship between paratyphoid diagnosis and anti-O antibody titres 19
Supplementary tables 20
Table S1. Number of unique participants experiencing each solicited symptom (of any severity) following vaccination 20
Table S2. Median and IQR of laboratory markers 7 days post-vaccination 21
Table S3. Grading of laboratory markers 7 days post vaccine 22
Table S4 – Serious Adverse events and AESIs in the trial 24
Table S5. Unsolicited adverse events 26
Table S6. Clinical and microbiological outcomes 27
Table S7. Antibody titres to *S.* Paratyphi A specific antigens post vaccination 29
Table S8. Antibody titres to *S.* Paratyphi A specific antigens post vaccination with two participants removed 30
Table S9. Vaccine Efficacy by analysis population 31

Table S10. Vaccine Efficacy by baseline anti-O IgG and interval between second vaccination and challenge 32
Table S11. Representativeness of study participants 33
References 34

VASP Study Team

Aley P[1,2], Albana X[6], Babbage G[6], Baughn H[1,2], Brito Mutunayagam[1,2] S, Brown C[7], Camara S[1,2], Chan S[3,4] Chisala W[5], Cifuentes Gutierrez L[1,2], Clutterbuck E[1,2], Comma E[1,2], Day N[1,2], De Masio C[1,2], Gill H[1,2], Hanumunthadu B[1,2], Huq R[3,4], Hyder-Wright A[3,4], Islam M[1,2], Jones E[1,2], Kanji N[1,2], Khan M[5], Mackay V[8], Mahmud R[1,2], Molina M[6], Morshead G[1,2], Mweu P[1,2], Mujaddi Y[1,2], Noristani R[1,2], Pantazi E[1,2], Payne RO[5], Pelia D[5], Penciu F[8], Phillips K[8], Presland L[6], Parker T[7], Reed T[6], Robinson H[1,2], Sahoo S[1,2], Shaw R,[1,2] Sherry E[7], Shrestha S[1,2], Singha A[1,2], Skidmore P[1,2], Smith W[1,2], Stuart A[1,2], Tester K[7], Tilzey G[8], Turnbull I[1,2], White R[1,2], Wright L[8], Yao A[1,2]

Institutions:

1. Oxford Vaccine Group, Department of Paediatrics, Centre for Clinical Vaccinology and Tropical Medicine, Churchill Hospital, Headington, Oxford, UK OX3 7LE
2. NIHR Oxford Biomedical Research Centre, Oxford, UK
3. Liverpool University Hospitals Foundation Trust
4. Liverpool School of Tropical Medicine, Liverpool, UK
5. Sheffield Teaching Hospitals NHS Foundation Trust, Sheffield, UK
6. NIHR Southampton Clinical Research Facility and Biomedical Research Centre, University Hospital Southampton NHS Foundation Trust;
7. NIHR/Wellcome Clinical Research Facility, University Hospitals Birmingham NHS Foundation Trust, Birmingham, UK
8. Bristol Medical School, University of Bristol

List of investigators

**Principal Investigators**	**Institution**
Professor Andrew Pollard (Chief Investigator)	Oxford Vaccine Group, University of Oxford, Oxford
Professor Brian Angus	Oxford Vaccine Group, University of Oxford & Oxford University Hospitals NHS Foundation Trust, Oxford

Dr Maheshi Ramasamy	Oxford Vaccine Group, University of Oxford & Oxford University Hospitals NHS Foundation Trust, Oxford
Professor Saul Faust	NIHR Southampton Clinical Research Facility and Biomedical Research Centre, University Hospital Southampton NHS Foundation Trust; and Faculty of Medicine and Institute for Life Sciences, University of Southampton, Southampton, UK
Dr Christopher Green	NIHR/Wellcome Clinical Research Facility, University Hospitals Birmingham NHS Foundation Trust, Birmingham, UK
Dr Rajeka Lazarus	Bristol Medical School, University of Bristol; UKHSA Southwest Regional Laboratory and Vaccine Development and Evaluation Centre, Bristol, UK
Dr Thomas Darton	Sheffield Teaching Hospitals NHS Foundation Trust
Dr Andrea Collins	Royal Liverpool and Broadgreen University Hospital Trust, Liverpool; , Liverpool School of Tropical Medicine,

DSMC members

**Name**	**Affiliation**
Chris Chiu	Imperial College London
George Bouliotis	University of Warwick
Paul Kaye	University of York
Celina Jin	Royal Melbourne Hospital
John Tregoning	Imperial College London
Tom Darton	*University of Sheffield*
Daniela Ferreira	*Liverpool School of Hygiene and Tropical Medicine*

Dr Thomas Darton and Prof Daniela Ferreira were initial members of the DSMC but were replaced by Dr John Tregoning and Dr Celina Jin when conflicts of interest arose due to changes in position. Prof Daniela Ferreria joined OVG in May 2022 and was therefore replaced by Dr John Tregoning in April 2022. Dr Thomas Darton was due to become a PI for the study in March 2023 and was therefore replaced by Dr Celina Jin in October 2022.

Supplementary methods

Inclusion Criteria

Participants must satisfy all the following criteria to be considered eligible for the study:

- Willing and able to give informed consent for participation in the study.
- Aged between 18 and 55 years inclusive at time of vaccination.
- In good health as determined by medical history, physical examination and clinical judgment of the study team.

- Willing to be available for all required appointments and if applicable to travel to vaccination and challenge site
- Agree (in the study team's opinion) to comply with all study requirements, including capacity to adhere to good personal hygiene and infection control precautions.
- Agree to allow study staff to contact their GP or equivalent NHS databases to access the participant's vaccination records, medical history and have their opinion solicited as to the participant's appropriateness for inclusion.
- Agree to allow study staff to access NHS health records and participant identifiable data as required for study purposes.
- Agree to allow their GP (and/or Consultant if appropriate), to be notified of participation in the study.
- Agree to allow UKHSA to be informed of their participation in the study.
- Agree to give their close household contacts written information informing them of the participants' involvement in the study and offering them voluntary screening for *S.* Paratyphi A carriage.
- Agree to have 24-hour contact with study staff during the four weeks post challenge and are able to ensure that they are contactable by mobile phone for the duration of the vaccination and challenge period until antibiotic completion.
- Have internet access to allow completion of the e-diary and real-time safety monitoring.
- Agree to avoid antipyretic/anti-inflammatory treatment from challenge until advised by a study doctor or until 14 days after challenge.
- Agree to refrain from donating blood for the duration of the study.
- Agree to provide their National Insurance/Passport number for the purposes of TOPS registration and for payment of reimbursement expenses.
- Participants must have received at least one dose of a SARS-CoV-2 vaccine that has been approved for use by the MHRA (or other national regulatory authority) $\geq$ four weeks prior to enrolment.
- Agree to not receive other vaccinations (e.g. Covid-19 vaccines) during the 7 days before and after study vaccination and during the 7 days before or 21 days post-challenge.

Exclusion Criteria

- The participant will not be enrolled if any of the following apply:
- History of significant organ/system disease that could interfere with trial conduct or completion. Including, for example, but not restricted to:
    - Cardiovascular disease including a diagnosis of hypertension
    - Respiratory disease
    - Haematological disease
    - Endocrine disorders
    - Renal or bladder disease, including history of renal calculi
    - Biliary tract disease, including biliary colic, asymptomatic gallstones or previous cholecystectomy

  - Gastro-intestinal disease including requirement for antacids, H_2-receptor antagonists, proton pump inhibitors or laxatives
  - Neurological disease
  - Metabolic disease
  - Autoimmune disease
  - Psychiatric illness requiring hospitalisation
  - Known or suspected drug misuse
  - Known or suspected alcohol misuse (alcohol misuse defined as an intake exceeding 42 units per week)
  - Infectious disease
- Have any known or suspected impairment of immune function, alteration of immune function, or prior immune exposure that may alter immune function to paratyphoid resulting from, for example:
  - Congenital or acquired immunodeficiency, including IgA deficiency
  - Human Immunodeficiency Virus infection or symptoms/signs suggestive of an HIV-associated condition
  - Receipt of immunosuppressive therapy such as anti-cancer chemotherapy or radiation therapy within the preceding 12 months or long-term systemic corticosteroid therapy.
- Receipt of immunoglobulin or any blood product transfusion within 3 months of study start.
- History of cancer (except squamous cell or basal cell carcinoma of the skin and cervical carcinoma in situ).
- HLA-B27 positive.
- Moderate or severe depression or anxiety as classified by the Hospital Anxiety and Depression Score at screening or challenge that is deemed clinically significant by the study doctors.
- Weight less than 50 kg.
- Presence of implants or prosthetic material.
- Anyone taking long-term medication (e.g. analgesia, anti-inflammatories or antibiotics) that may affect symptom reporting or interpretation of the study results.
- Contraindication to fluoroquinolones, macrolide antibiotics, co-trimoxazole or ceftriaxone.
- Family history of aneurysmal disease
- Participants of childbearing potential who are pregnant, lactating or who are unwilling to ensure that they or their partner use effective contraception 30 days prior to vaccination and continue to do so until three negative stool samples have been obtained at least one week after completion of antibiotic treatment.
- Full-time, part-time or voluntary occupations involving:
  - Clinical or social work with direct contact with young children (defined as those attending pre-school groups or nursery or aged under 2 years), or

    - Clinical or social work with direct contact with highly susceptible patients or persons in whom typhoid infection would have particularly serious consequences
    - Commercial food handling (involving preparing or serving unwrapped foods not subjected to further heating)
      (unless willing to avoid work from vaccination until demonstrated not to be infected with *S*. Paratyphi A after challenge by clearance samples in accordance with guidance from UKHSA and willing to allow study staff to inform their employer).
- Close household contact with:
    - Young children (defined as those attending pre-school groups, nursery or those aged less than 2 years)
    - Individuals who are immunocompromised (including pregnancy).
- Scheduled elective surgery or other procedures requiring general anaesthesia during the study period.
- Participants who have participated in another research study involving an investigational product that might affect risk of paratyphoid infection or compromise the integrity of the study within the 30 days prior to enrolment (e.g. significant volumes of blood already taken in previous study).
- Detection of any abnormal results from screening investigations (at the clinical discretion of the study team).
- Inability to comply with any of the study requirements (at the discretion of the study staff and the participant's General Practitioner).
- Any other social, psychological or health issues which, in the opinion of the study staff, may
    - put the participant or their contacts at risk because of participation in the study,
    - adversely affect the interpretation of the primary endpoint data,
    - Impair the participant's ability to participate in the study.
- Have any history of allergy to vaccine/placebo components
- Having been resident in an enteric fever endemic country for 6 months or more.
- Have previously been diagnosed with laboratory-confirmed typhoid or paratyphoid infection or been given a diagnosis compatible with enteric fever.
- Have participated in previous typhoid or paratyphoid challenge studies (with ingestion of challenge agent).
- Have received any oral typhoid vaccination (e.g. Ty21a or M01ZH09) at any time.
- Have a prolonged corrected QT interval (>450 milliseconds) on ECG screening.
- Significant blood donation or planned blood donation prior to enrolment.

Temporary Exclusion at Vaccination

Participants will be temporarily excluded from receiving vaccination if presenting at a vaccination visit with the following:

- Acute gastrointestinal illness within 24-hours prior to vaccination.
- Significant infection within the previous 7 days.

- Participant has experienced fever (>37.5°C) or subjective febrile symptoms within the previous 3 days (even with a negative COVID-19 test).
- Symptoms of COVID-19, or confirmed infection (according to government guidelines) within the 5 days prior to vaccination visit; as per guidance in Section 9.19
- History of any systemic antibiotic therapy during the previous 5 days.
- Any systemic corticosteroid (or equivalent) treatment in the previous 14 days, or for more than seven consecutive days within the past 3 months).
- Receipt of another enteric live vaccine within 4 weeks prior to vaccination or an injected live or killed vaccine within 7 days prior to vaccination.
- Plan to receive any vaccine other than the study vaccine within 7 days following vaccination.
- Therapy with antacids, proton pump inhibitors or H_2-receptor antagonists within 24 hours prior to vaccination.
- Unavailable for post-vaccination visits, second vaccination visit, and challenge visit.

Temporary Exclusion at Challenge

Participants will be temporarily excluded from challenge if presenting at the challenge visit with the following. Participants can be challenged up to 28 days (and longer in exceptional circumstances) after their original challenge date.

- Significant acute or acute-on-chronic infection within the previous 7 days or have experienced fever (>37.5°C) or subjective febrile symptoms within the previous 3 days (even with a negative COVID-19 test).
- Symptoms of COVID-19, or confirmed infection (according to government guidelines) within 5 days prior to challenge visit; as per guidance in Section 9.19
- History of any systemic antibiotic therapy during the previous 5 days.
- Any systemic corticosteroid (or equivalent) treatment in the previous 14 days, or for more than seven consecutive days within the past 3 months.
- Therapy with antacids, proton pump inhibitors or H_2-receptor antagonists within 24 hours prior to challenge.
- Participant has not received two doses of the study vaccine/placebo.
- Anaemia felt to be clinically significant by the study team.
- Receipt of a COVID-19 vaccine in preceding 7 days

Secondary outcomes for the study

	Objective	Outcome
Secondary	a. To compare the clinical and laboratory features of the host responses following challenge with *Salmonella* Paratyphi A (strain NVGH308) in	Comparison of the clinical course of paratyphoid infection after challenge between placebo and CVD 1902 groups, in particular:<br>• time to onset of symptoms<br>• duration of illness

	participants vaccinated with CVD 1902 compared to placebo, including the time course of illness, development of bacteraemia and the inflammatory response.	• symptom severity<br>• time to onset of bacteraemia<br>• time to onset of stool shedding<br>• inflammatory response<br>Using clinical reporting, physical examination findings, microbiological assays to detect *S.* Paratyphi A in blood and stool, and laboratory assays to monitor inflammatory responses.
	b. To compare the host immune response following vaccination with CVD 1902, compared with placebo including innate, antibody and cell-mediated responses and persistence of immunity and to relate these responses to the protective effect of vaccination.	Immunological laboratory assays to assess innate, humoral, cell-mediated and mucosal responses to vaccination at baseline (Day -42) and post-vaccination time points, these may include:<br>• *S.* Paratyphi A antigen specific antibodies and serum bactericidal antibody titres<br>• Cell-mediated responses (including antigen specific cell frequencies, description of lymphocyte populations, B and T cell repertoire)<br>• Cytokine and acute phase reactant profile and kinetics
	c. To compare the host immune response following *S.* Paratyphi A challenge following vaccination with CVD 1902 or placebo.	Immunological laboratory assays to assess innate, humoral, cell-mediated and mucosal responses to challenge will be taken at various time points following challenge (for time points see section 7.9, Table 3b and section 7.10, Table 3c) in a variety of sample types, these may include:<br>• Cell-mediated responses (including antigen specific cell frequencies, description of lymphocyte populations, B and T cell repertoire)<br>• Cytokine and acute phase reactant profile and kinetics<br>• *S.* Paratyphi A antigen-specific IgA, IgM and IgG antibodies

	d. To assess the safety and tolerability of CVD 1902	Clinical observation and participant recording of symptoms, both solicited and unsolicited plus safety laboratory data
	e. To investigate immunological correlates of protection for *S*. Paratyphi A infection	Immunological response data post-vaccination (including *S*. Paratyphi A specific antibody titres, cell-mediated responses) will be combined with vaccine efficacy data following *S.* Paratyphi A challenge to investigate if particular immunological markers could be used to predict protection from paratyphoid infection

Enzyme-linked immunosorbent assay (ELISA) for *S.* Paratyphi A antigens

96-well Greiner plates were coated with either 5ug/mL of COPS antigen (supplied by University of Maryland) or 2ug/mL of FliC antigen (supplied by University of Maryland) diluted in phosphate buffered saline (pH 7.2) and incubated overnight at 4°C for 18 hours. Plates were then washed five times with phosphate buffered saline containing 0.05% Tween (PBST) and blocked for 2 hours at 37°C with 5% non-fat milk powder diluted with PBST (NFMP-PBST). Samples were thawed and diluted in NFPM-PBST. Plates were washed five times and samples added in triplicate to the plates alongside 3 positive internal controls (created from pooled serum at day 90 from individuals in a prior *S.* Paratyphi A challenge study) and one negative control (created from known negative pooled serum pre-challenge from the same study) and eight two-fold serial dilutions of the standard serum. The standard serum was created from a pool of individuals exposed to the live typhoid vaccine M01ZH09 supplied by Emergent BioSolutions and the associated standard curve was assigned arbitrary ELISA units. Plates were incubated for an hour at 37°C then washed five times with PBST. Secondary antibody (goat anti-human IgG or IgA conjugated to horseradish peroxide) was diluted in NFMP-PBST at 1 in 20,000 (IgG) or 1 in 1,000 (IgA) and then added to the plates which were incubated at 37 °C for 1 hour. Following a further five washes with PBST 3,3’, 5, 5’ Tetramethylbenzidine was added to develop the plates followed by 2 M sulphuric acid to stop the reaction. Plates were read using an ELx808 microplate reader (BioTek Instruments, Winooski, VT, USA) at 450nm and 630nm. Standardised ELISA units (EUs) for each sample were calculated from the concentration obtained from the standard curve multiplied by dilution factor, using BioTeK Gen5 software version 3.09.

Vaccination e-diaries

Participants self-graded the following solicited symptoms in the vaccination e-diary every day for 7 days (Day 0 to Day 6) following prime and boost vaccination. For purposes of summarising and

describing adverse events the following translations were used; 1= mild, 2 = moderate, 3 = severe and 4 = life-threatening.

	0	1	2	3	4
**Nausea/Vomiting**	No symptoms	Present but no interference with activity or 1 – 2 episodes in 24 hours	Some interference with activity or more than 2 episodes in 24 hours	Significant; prevents daily activity	Emergency department visit or hospitalisation
**Diarrhoea**	No symptoms	3-4 loose stools in 24 hrs	5-6 loose stools in 24 hrs	7 or more loose stools in 24 hrs	Emergency department visit or hospitalisation
**Eating less than usual or loss of appetite**	No symptoms	Eat less than normal for 1-2 meals	Miss 1-2 meals completely	Miss all meals	Emergency department or hospital visit required
**Generally unwell**	No symptoms	Present but no interference with activity	Some interference with activity	Significant; prevents daily activity	Emergency department visit or hospitalisation
**Abdominal/ stomach pain**	No symptoms	Present but no interference with activity	Some interference with activity	Significant; prevents daily activity	Emergency department visit or hospitalisation
**Headache**	No symptoms	Present but no interference with activity	Some interference with activity	Significant; prevents daily activity	Emergency department visit or hospitalisation

Challenge e-diaries

Participants self-graded each of the following symptoms in the challenge e-diary every day for 21 days following challenge administration. For purposes of summarising and describing adverse events the following translations were used; 1= mild, 2 = moderate, 3 = severe and 4 = life-threatening.

	0	1	2	3	4
**Headache**	No symptoms	Present but no interference with activity	Some interference with activity	Significant; any use of codeine phosphate or prevents daily activity	Emergency department visit or hospitalisation

**Generally unwell**	No symptoms	Present but no interference with activity	Some interference with activity	Significant; prevents daily activity	Emergency department visit or hospitalisation
**Eating less than usual or loss of appetite**	No symptoms	Eat less than normal for 1-2 meals	Miss 1-2 meals completely	Miss all meals	Emergency department or hospital visit required
**Abdominal/ stomach pain**	No symptoms	Present but no interference with activity	Some interference with activity	Significant; any use of codeine phosphate or prevents daily activity	Emergency department visit or hospitalisation
**Nausea/vomiting**	No symptoms	Present but no interference with activity or 1 – 2 episodes in 24 hours	Some interference with activity or more than 2 episodes in 24 hours	Significant; prevents daily activity	Emergency department visit or hospitalisation
**Muscle pain**	No symptoms	Present but no interference with activity	Some interference with activity	Significant; any use of codeine phosphate or prevents daily activity	Emergency department visit or hospitalisation
**Joint pain**	No symptoms	Present but no interference with activity	Some interference with activity	Significant; any use of codeine phosphate or prevents daily activity	Emergency department visit or hospitalisation
**Cough**	No symptoms	Present but no interference with activity	Some interference with activity	Significant; any use of codeine phosphate or prevents daily activity	Emergency department visit or hospitalisation
**Diarrhoea**	No symptoms	3-4 loose stools in 24 hrs	5-6 loose stools in 24 hrs	7 or more loose stools in 24 hrs	Emergency department visit or hospitalisation
**Constipation**	No symptoms	Present but no interference with activity or 1 – 2 episodes in 24 hours	Some interference with activity or more than 2 episodes in 24 hours	Significant; prevents daily activity	Emergency department visit or hospitalisation
**Rash**	No symptoms	Yes/No			

Antibiotic treatment

- 1st line: Oral ciprofloxacin 500mg twice daily for 14 days (the first 21 patients in the study received 7 days of ciprofloxacin as per protocol and clinical guidance but the course was increased to 14 days after noting a small increase in number of positive convalescent stool samples)
- 2nd line: Oral trimethoprim/sulfamethoxazole 160/800 mg twice daily for 7-14 days or azithromycin 1g stat dose followed by 500mg daily for 6-13 days.

- 3rd line: Oral amoxicillin 500mg TDS for 7-14 days.

Definition of severe paratyphoid fever

Severe paratyphoid fever is diagnosed if ANY of the following apply
**Oral temperature $\geq$ 40.0°C**
**Systolic blood pressure $\leq$ 85 mmHg**
**Significant lethargy or confusion**
**Gastrointestinal bleeding**
**Gastrointestinal perforation**
**Any grade 4 or above laboratory abnormality, as defined in Appendix C: Grading the severity of laboratory Adverse Events**

Supplementary figures

Figure S1. Symptoms of diagnosed participants during challenge

A

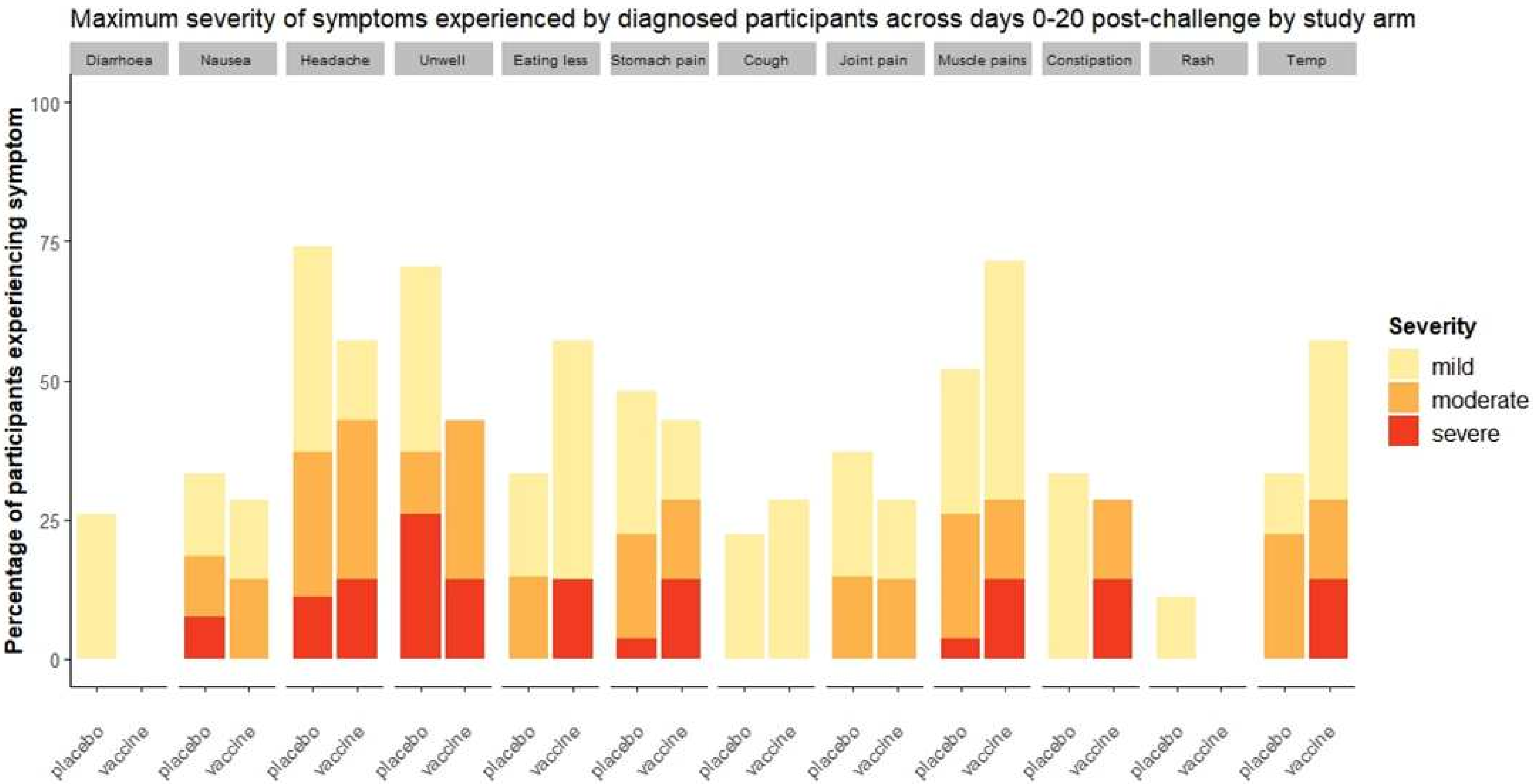

B

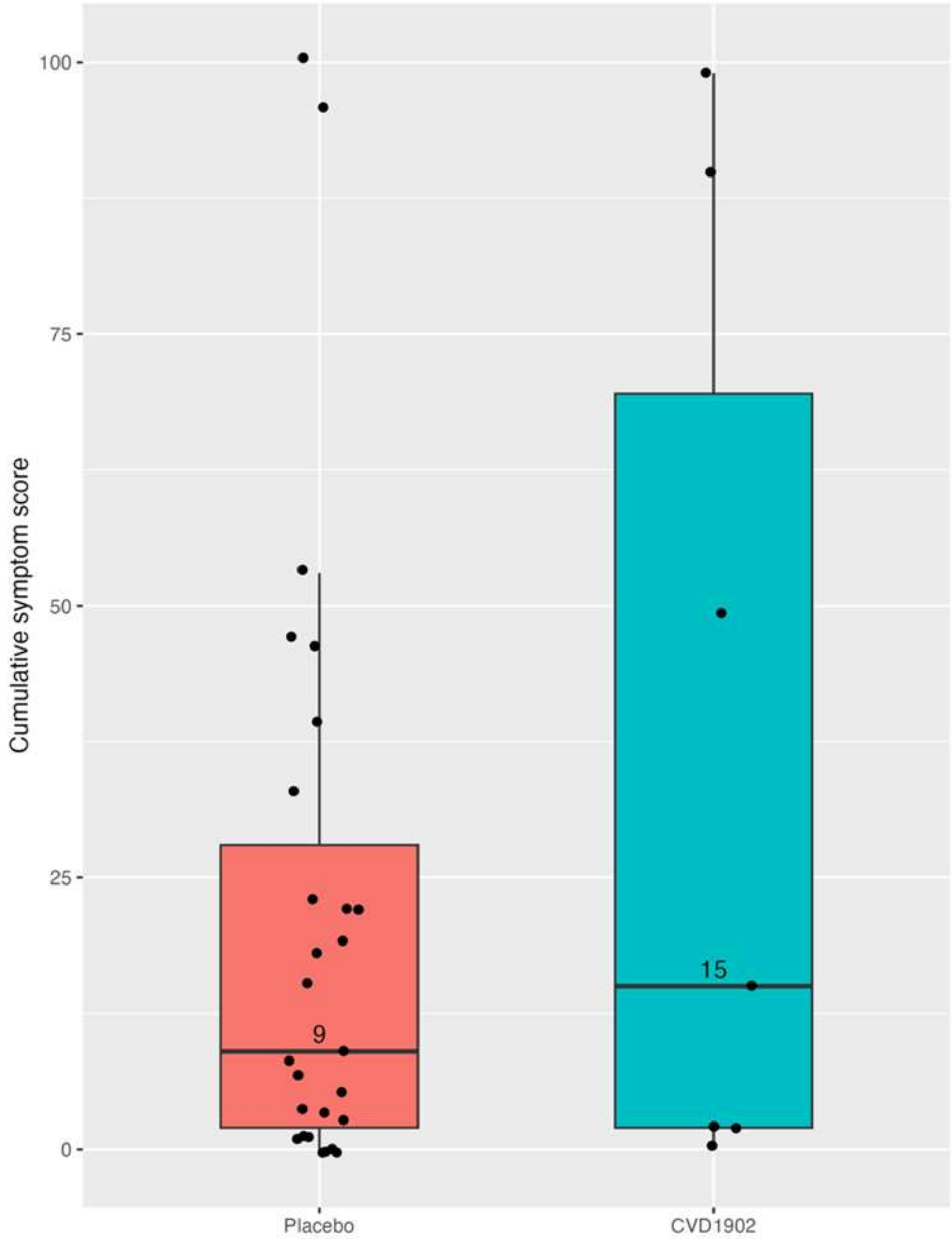

Figure S1

- *A- Bar chart of maximum severity of each self-reported solicited symptom experienced by diagnosed participants from day 0 -20 following challenge.*
- *B- Boxplot showing cumulative symptom scores of all self-reported solicited symptoms in diagnosed participants from day 0-20 following challenge.*

Figure S2. Microbiological outcomes during challenge.

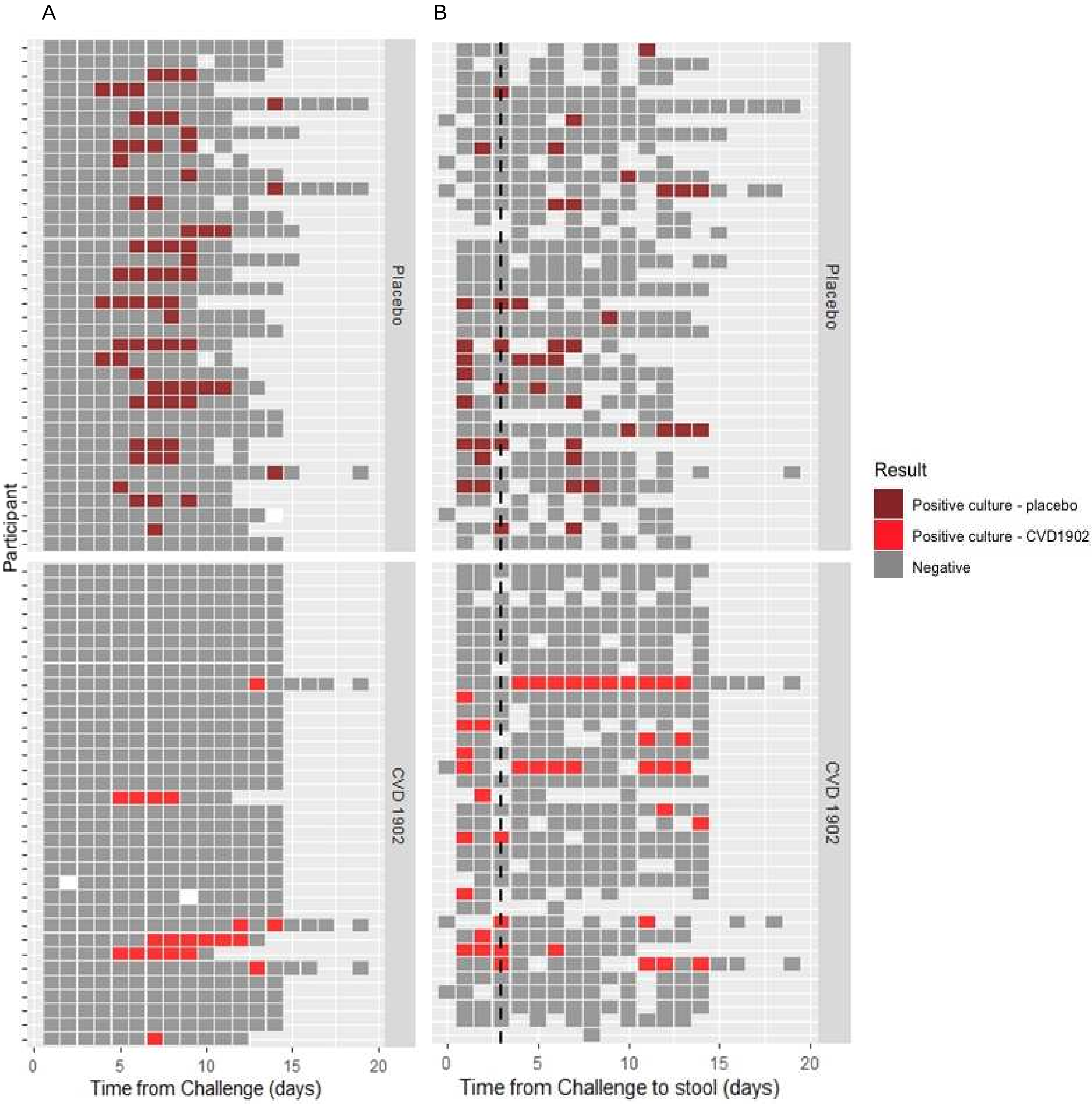

Figure S2. Tile plot showing the pattern of bacteraemia (A) and stool shedding (B) in all participants during challenge. Coloured squares represent a positive culture on that day and dark grey represent negative cultures. Blank (light grey) squares represent days where no result was available (usually due to sample not being taken). Dashed black line represents 72 hours (day 3) following challenge, as prior to this positive stool cultures were not counted as shown in Figure S3 and Table S6.

Figure S3. Stool culture positivity during challenge between groups

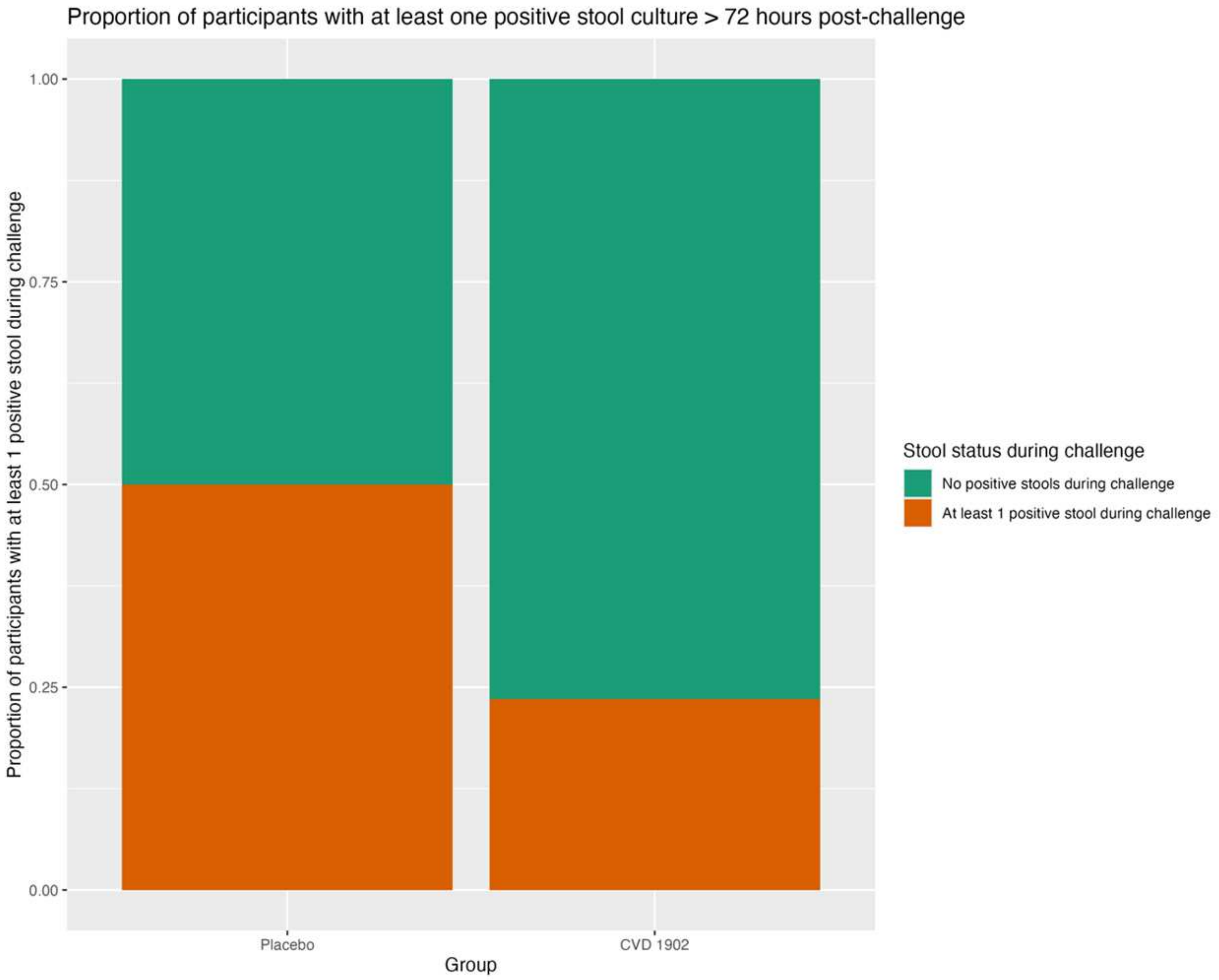

Figure S3. Proportion of all participants, by vaccine group, with at least one positive stool culture from 72 hours post-challenge to last challenge visit (either day 14 post-challenge if not diagnosed or 4 days post-diagnosis if diagnosed).

Figure S4: Anti-FliC IgG (A) and IgA (B) antibody response following vaccination

A

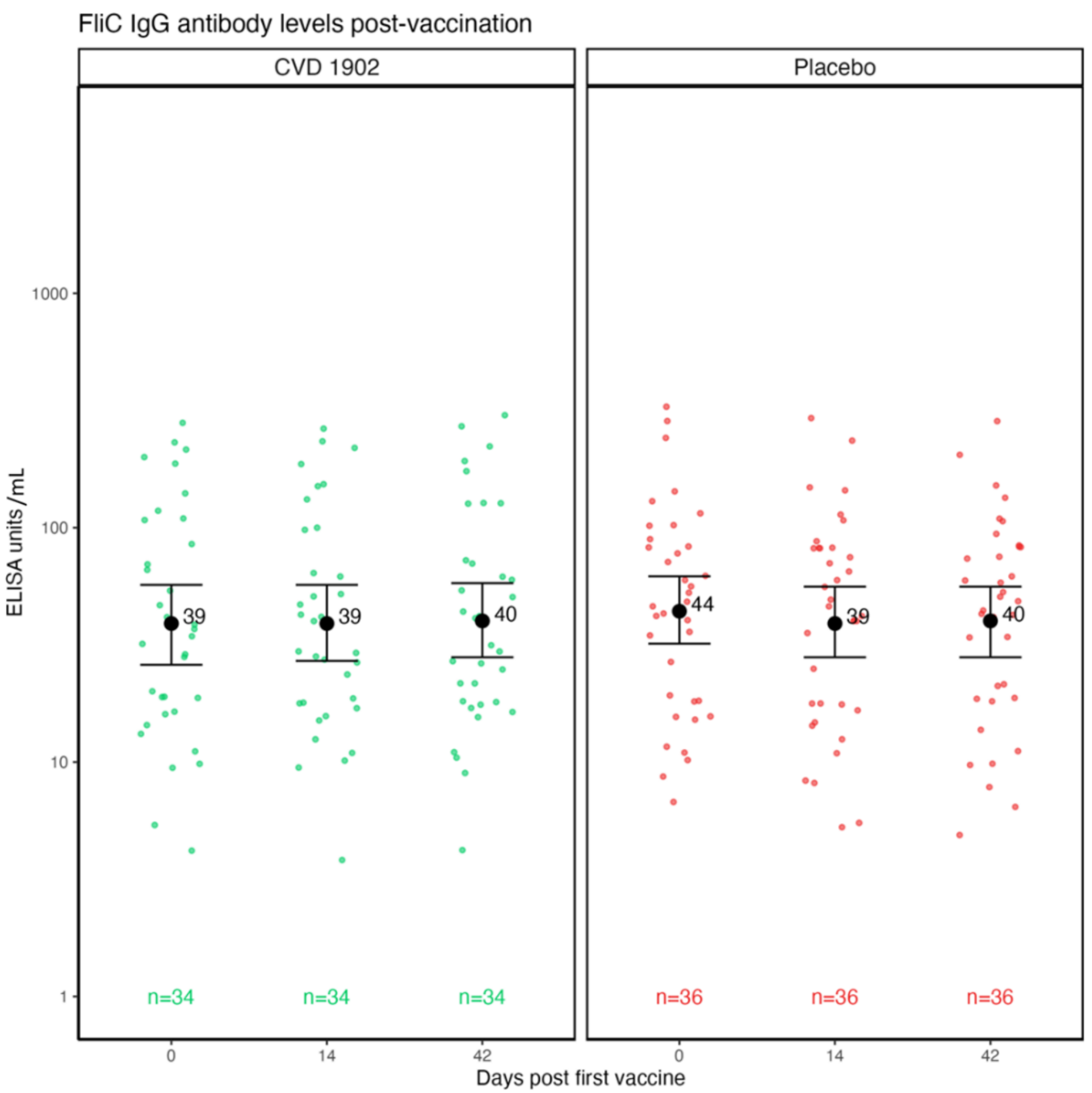

B

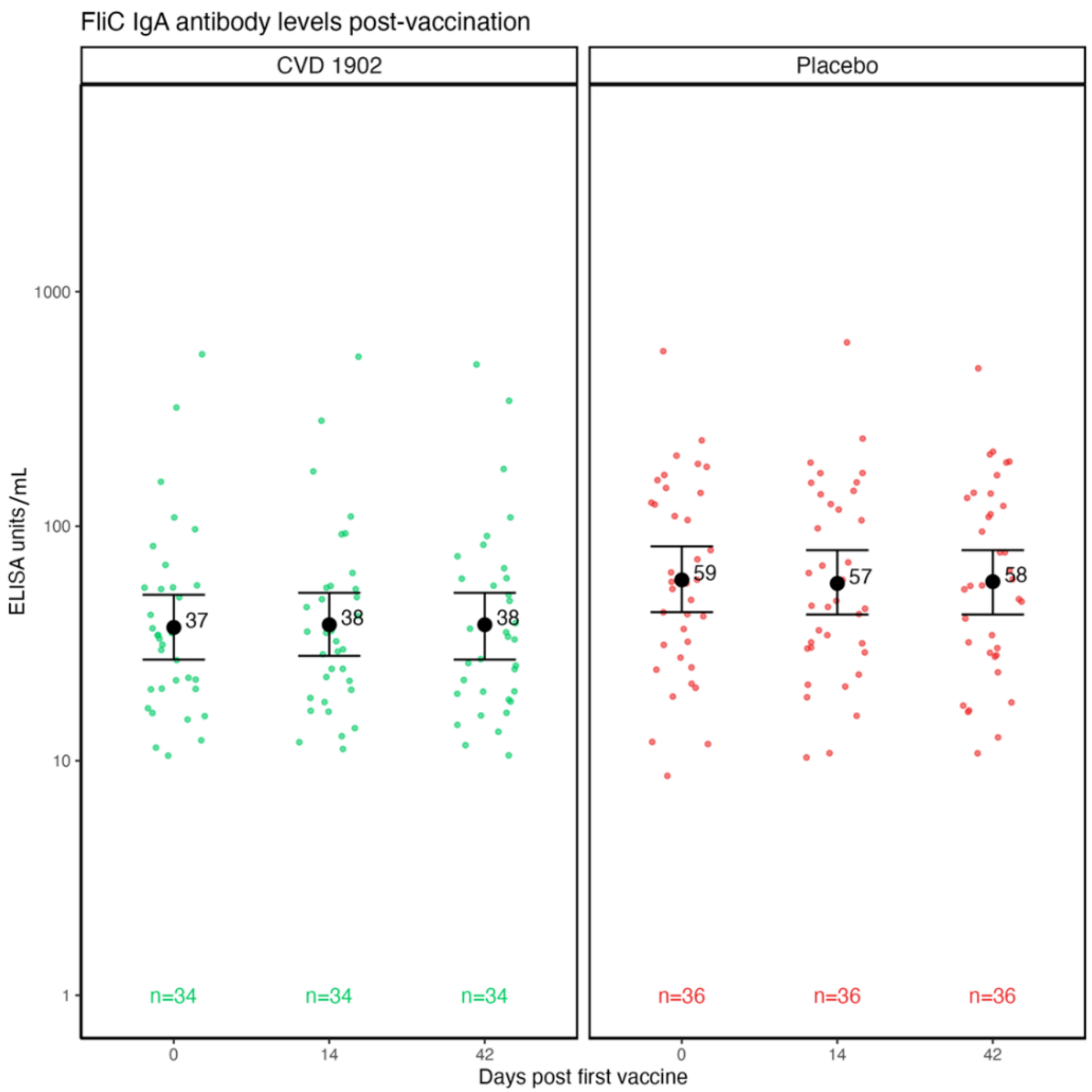

Figure S4. Anti-SPA FliC antigen titres measured following at baseline, 14 days post-first vaccine and 42 days post second vaccine (28 days post second vaccine). Individual dots represent individual participant values and error bars represent the mean geometric mean titres and 95% confidence interval for each timepoint. Numbers above error bars are the mean GMT for each timepoint. Plot A) represents anti-O IgG responses and plot B) represents anti-O IgA response.

Figure S5. Antibody titres at D42 between protected and susceptible groups

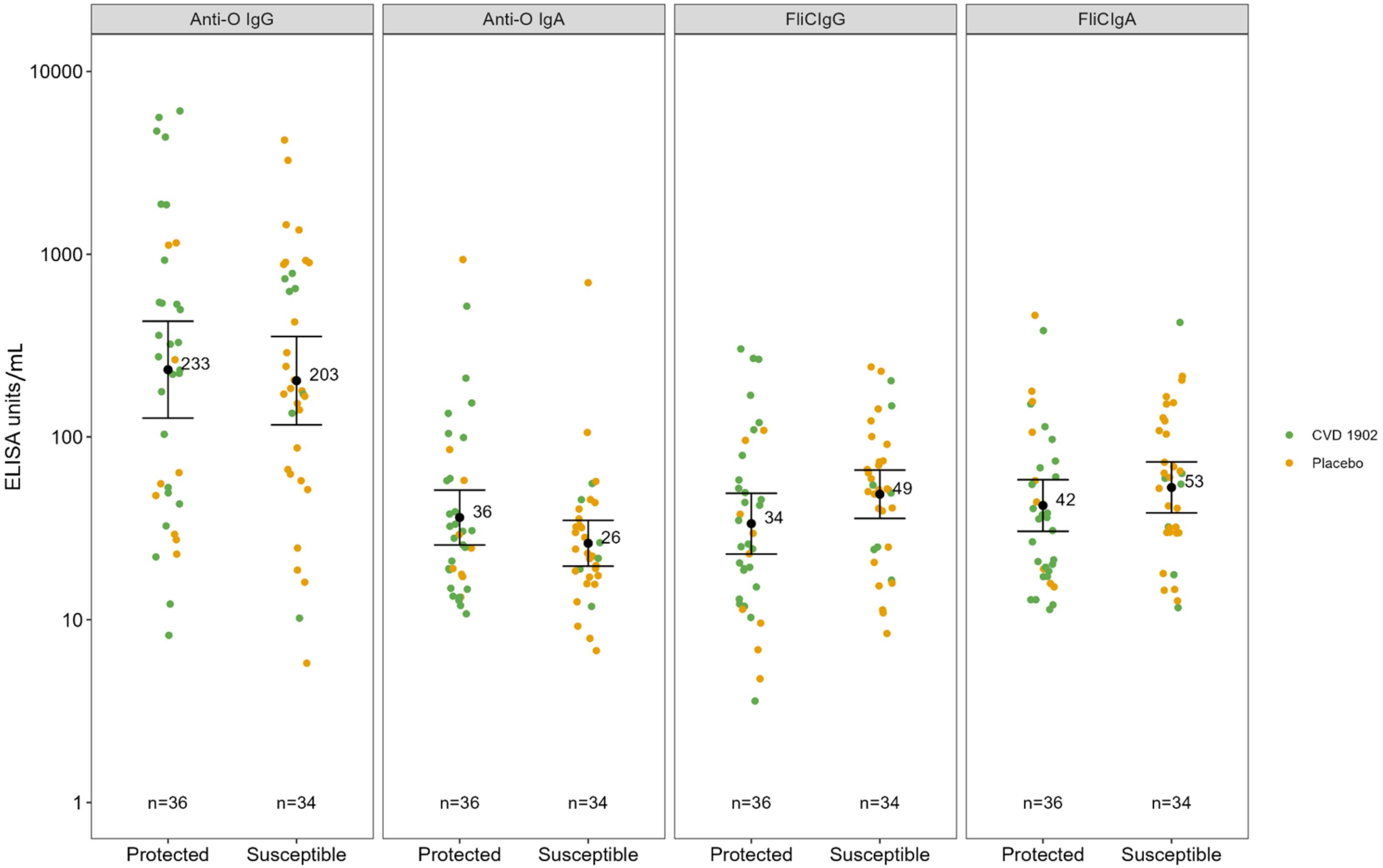

Figure S5. Antibody titres immediately pre-challenge (i.e. 42 days post first-vaccine, 28 days post second-vaccine) compared between those that went on to be protected from paratyphoid and those that were susceptible to paratyphoid diagnosis. Individual dots represent individual participant values and error bars represent the mean geometric mean titres and 95% confidence interval for each group. Numbers by error bars are the mean GMT for group.

Figure S6. Logistic regression modelling of relationship between paratyphoid diagnosis and anti-O antibody titres
A - IgG; B - IgA

A

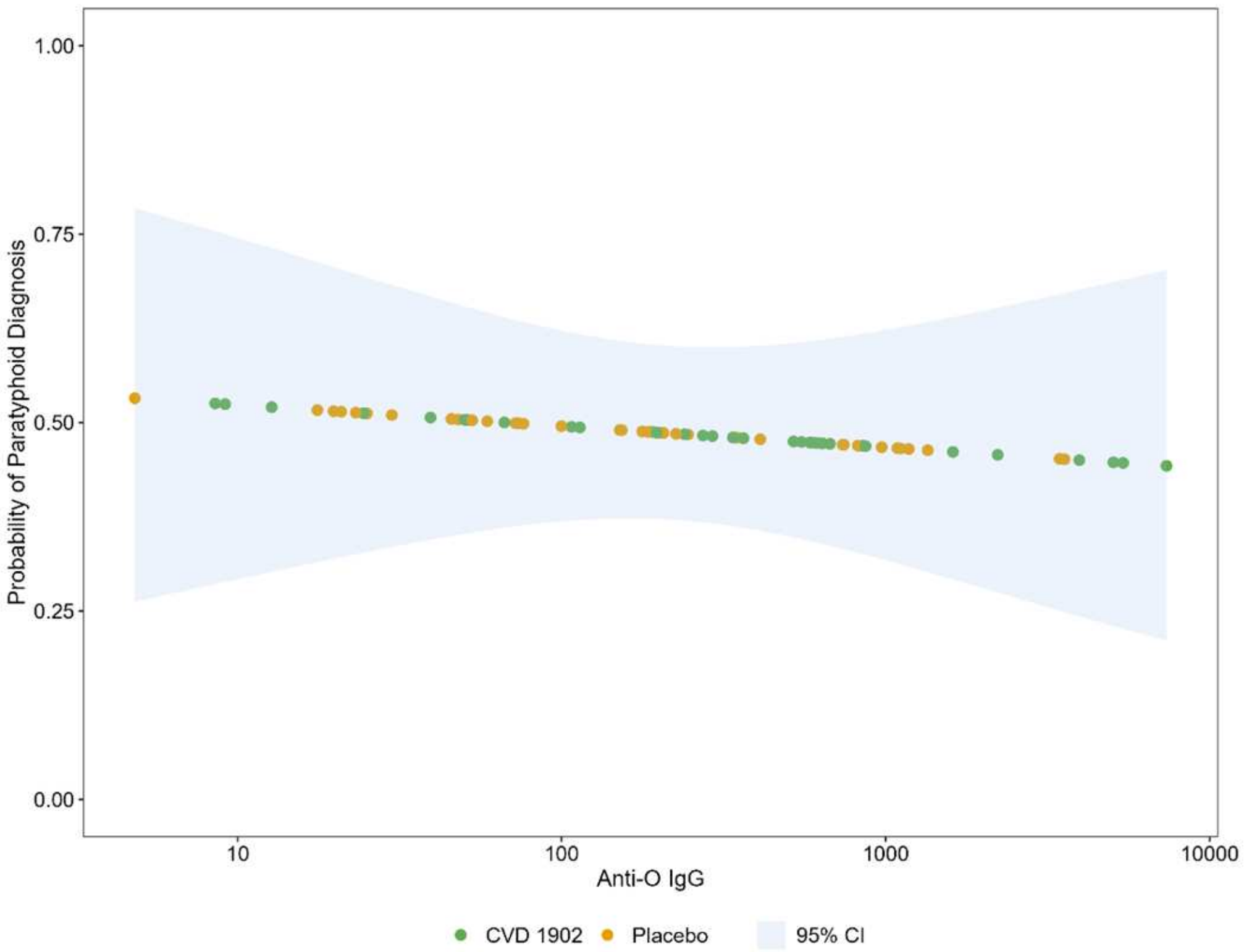

B

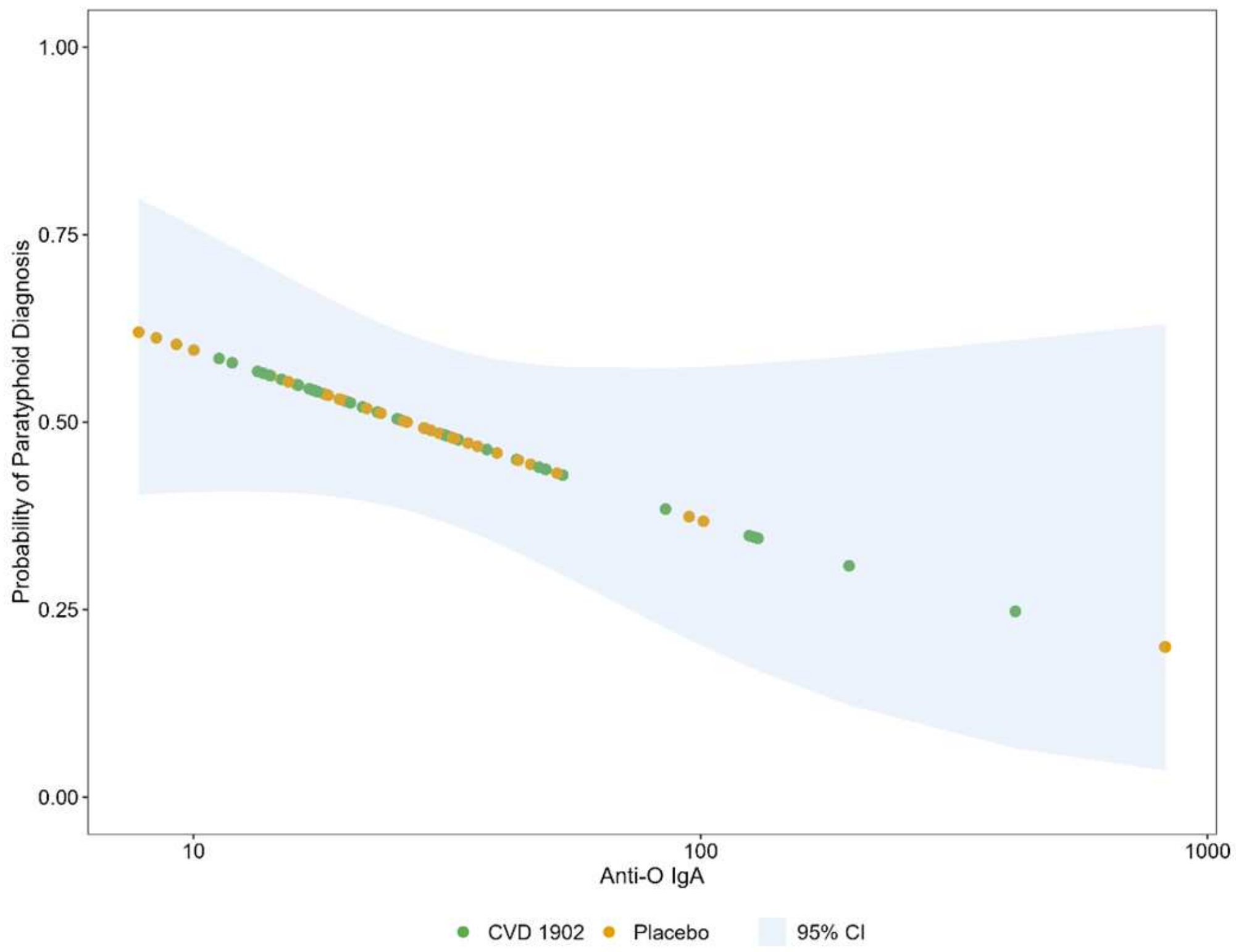

Supplementary tables

Table S1. Number of unique participants experiencing each solicited symptom (of any severity) following vaccination

**First vaccine**	**CVD 1902 group (n = 35)**	**Placebo group (n= 37)**
Headache	15 (43%)	15 (40.5%)
Nausea/vomiting	6 (17%)	0
Diarrhoea	3 (9%)	4 (11%)
Generally unwell	12 (34%)	3 (8%)
Eating less	9 (26%)	5 (13.5%)
Stomach pain	11 (31%)	9 (24%)
Temperature	2 (6%)	1 (3%)
**Second vaccine**	**CVD 1902 group (n = 34)**	**Placebo group (n= 36)**
Headache	8 (23.5%)	10 (28%)
Nausea/vomiting	9 (26%)	5 (14%)
Diarrhoea	4 (12%)	5 (14%)
Generally unwell	10 (29%)	9 (25%)
Eating less	8 (23.5%)	5 (14%)
Stomach pain	6 (18%)	3 (8%)
Temperature	0	2 (5.5%)

Table S2. Median and IQR of laboratory markers 7 days post-vaccination

	First vaccine		Second vaccine	
	**CVD 1902 group**	**Placebo group**	**CVD 1902 group**	**Placebo group**
**Sodium**	139 (139 -140)	139 (138-140)	139 (138-140)	139 (137-141)
**Potassium**	4.1 (3.9-4.3)	4.1 (3.8-4.3)	4.1 (3.8-4.2)	4.0 (3.8-4.2)
**Creatinine**	72 (59.2 – 78)	73 (66-84)	71 (57-79)	73 (65-85)
**ALT**	18 (15.2 -24.8)	17 (13-23)	19 (15-26)	15.5 (13-20)
**C-reactive protein**	1 (0.7-1.58)	1 (0.8-2.5)	1 (0.6-1.7)	1 (0.6-1.5)
**Haemoglobin**	140 (132-148)	142 (135-152)	140.5 (130-146)	138 (130-148.5)
**White-cell count**	5.22 (4.45-6.01)	6.04 (5.26-6.95)	5.44 (4.72-6.10)	6.09 (5.15-7.15)
**Neutrophil count**	2.94 (2.34 -3.78)	3.48 (2.99-4.11)	3.12 (2.60 – 3.12)	3.48 (2.95 – 3.49)
**Platelets**	254 (230-286)	248 (217-312)	260.5 (229-291)	246 (218-296)

Values shown as median (IQR)

Table S3. Grading of laboratory markers 7 days post vaccine

	Prime +6 days		Boost +6 days	
**Biochemistry marker**<br>Self-reported grade	**Placebo**<br>N = 37[1]	**Vaccine**<br>N = 34[1]	**Placebo**<br>N = 36[1]	**Vaccine**<br>N = 33[1]
**Inc. Sodium**				
0	37 (100%)	34 (100%)	35 (97%)	33 (100%)
1-2	0 (0%)	0 (0%)	1 (2.8%)	0 (0%)
3-4	0 (0%)	0 (0%)	0 (0%)	0 (0%)
**Dec. Sodium**				
0	37 (100%)	34 (100%)	36 (100%)	33 (100%)
1-2	0 (0%)	0 (0%)	0 (0%)	0 (0%)
3-4	0 (0%)	0 (0%)	0 (0%)	0 (0%)
**Inc. Potassium**				
0	37 (100%)	33 (97%)	36 (100%)	33 (100%)
1-2	0 (0%)	1 (2.9%)	0 (0%)	0 (0%)
3-4	0 (0%)	0 (0%)	0 (0%)	0 (0%)
**Dec. Potassium**				
0	37 (100%)	34 (100%)	35 (97%)	33 (100%)
1-2	0 (0%)	0 (0%)	1 (2.8%)	0 (0%)
3-4	0 (0%)	0 (0%)	0 (0%)	0 (0%)
**Urea**				
0	35 (95%)	33 (97%)	36 (100%)	32 (97%)
1-2	2 (5.4%)	1 (2.9%)	0 (0%)	1 (3.0%)
3-4	0 (0%)	0 (0%)	0 (0%)	0 (0%)
**Creatinine**				
0	37 (100%)	34 (100%)	36 (100%)	33 (100%)
1-2	0 (0%)	0 (0%)	0 (0%)	0 (0%)
3-4	0 (0%)	0 (0%)	0 (0%)	0 (0%)
**CRP**				
0	36 (97%)	33 (97%)	35 (97%)	32 (97%)
1-2	1 (2.7%)	1 (2.9%)	1 (2.8%)	1 (3.0%)
3-4	0 (0%)	0 (0%)	0 (0%)	0 (0%)
**Bilirubin**				
0	36 (97%)	32 (94%)	34 (94%)	32 (97%)
1-2	1 (2.7%)	2 (5.9%)	2 (5.6%)	1 (3.0%)
3-4	0 (0%)	0 (0%)	0 (0%)	0 (0%)
**ALT**				
0	36 (97%)	32 (94%)	36 (100%)	32 (97%)
1-2	1 (2.7%)	2 (5.9%)	0 (0%)	1 (3.0%)
3-4	0 (0%)	0 (0%)	0 (0%)	0 (0%)
**ALP**				
0	37 (100%)	34 (100%)	36 (100%)	33 (100%)
1-2	0 (0%)	0 (0%)	0 (0%)	0 (0%)
3-4	0 (0%)	0 (0%)	0 (0%)	0 (0%)
**Albumin**				
0	37 (100%)	34 (100%)	36 (100%)	33 (100%)
1-2	0 (0%)	0 (0%)	0 (0%)	0 (0%)
3-4	0 (0%)	0 (0%)	0 (0%)	0 (0%)

**Full blood count marker**<br>Self-reported grade	**Placebo**<br>N = 37[1]	**Vaccine**<br>N = 34[1]	**Placebo**<br>N = 36[1]	**Vaccine**<br>N = 34[1]
**Haemoglobin Dec.**				
0	34 (92%)	31 (91%)	28 (78%)	28 (82%)
1-2	3 (8.1%)	3 (8.8%)	8 (22%)	6 (18%)
3-4	0 (0%)	0 (0%)	0 (0%)	0 (0%)
**WBC Inc.**				
0	37 (100%)	34 (100%)	36 (100%)	33 (97%)
1-2	0 (0%)	0 (0%)	0 (0%)	1 (2.9%)
3-4	0 (0%)	0 (0%)	0 (0%)	0 (0%)
**WBC Dec.**				
0	37 (100%)	34 (100%)	36 (100%)	33 (97%)
1-2	0 (0%)	0 (0%)	0 (0%)	1 (2.9%)
3-4	0 (0%)	0 (0%)	0 (0%)	0 (0%)
**Platelets Dec.**				
0	36 (97%)	34 (100%)	36 (100%)	34 (100%)
1-2	1 (2.7%)	0 (0%)	0 (0%)	0 (0%)
3-4	0 (0%)	0 (0%)	0 (0%)	0 (0%)
**Neutrophils Dec.**				
0	35 (95%)	31 (91%)	34 (94%)	32 (94%)
1-2	2 (5.4%)	3 (8.8%)	2 (5.6%)	2 (5.9%)
3-4	0 (0%)	0 (0%)	0 (0%)	0 (0%)

[1] n (%)

Table S4 – Serious Adverse events and AESIs in the trial

**SAE**										
**ID**	**Study Arm**	**Stage of study**	**Classification**	**Diagnosis**	**Related to vaccine?**	**Related to challenge?**	**Related to other medication?**	**Outcome**	**Days since challenge**	**SAE duration in days**
12	Placebo	Post-challenge	Important medical event	Hospital day-admission for IV ceftriaxone to treat S. Paratyphi A relapse	No	Yes	No	Recovered	40	22
17	CVD 1902	Post-challenge	Significant disability	Achilles tendon rupture	No	No	Yes	Recovered	153	242
36	CVD 1902	Post-challenge	Hospitalisation	Gastroenteritis requiring IV fluids	No	No	No	Recovered	283	7
60	Placebo	Post-challenge	Hospitalisation	Possible repeat S. Paratyphi A relapse	No	Yes	No	Recovered	72	88
**AESI**										
**ID**	**Study Arm**	**Stage of study**	**Severity**	**Diagnosis**	**Related to vaccine?**	**Related to challenge?**	**Related to other medication?**	**Ongoing**	**Days since challenge**	**AESI duration in days**
7**	Placebo	Post-challenge	Mild	Progression to convalescent shedding of bacteria (defined as positive stool culture at least 1 week after completion of second course of antibiotics)	No	Yes	No	No	21	152
12	Placebo	Post-challenge	Moderate	Relapse of paratyphoid infection (defined as recurrence of confirmed paratyphoid infection following successful treatment)	No	Yes	No	Upgraded to a SAE	34	6
22**	Placebo	Post-challenge	Mild	Progression to convalescent shedding of bacteria (defined as positive stool culture at least 1 week after completion of second course of antibiotics)	No	Yes	No	No	33	102
60*	Placebo	Post-challenge	Mild	Relapse of paratyphoid infection (defined as recurrence of confirmed paratyphoid infection following successful treatment)	No	Yes	No	No	38	16

60*	Placebo	Post-challenge	Requires emergency or hospital visit	Relapse of paratyphoid infection (defined as recurrence of confirmed paratyphoid infection following successful treatment)	No	Yes	No	No	72	88
65	Placebo	Post-challenge	Severe	Relapse of paratyphoid infection (defined as recurrence of confirmed paratyphoid infection following successful treatment)	No	Yes	No	No	41	19

**One participant had 2 episodes of relapse (see Table S6).*

*** Two participants had a further episode of positive stool following re-treatment (see Table S6).*

Table S5. Unsolicited adverse events

	Placebo group (n = 37[1])	CVD 1902 group (n = 35[1])
**Number of unique participants with at least one AE**	33 (89.2%)	31 (88.6%)
**Total number of Adverse Events**	103	101
**Timing**		
Vaccination	28 (27%)	34 (34%)
Post-challenge	75 (73%)	67 (66%)
**Severity**		
Mild	62 (60%)	64 (63%)
Moderate	27 (26%)	30 (30%)
Severe	13 (13%)	7 (6.9%)
Requires emergency or hospital visit	1 (1.0%)	0 (0%)
**Causality to study vaccine**		
Not-related	100 (97%)	92 (91%)
Possible	2 (1.9%)	7 (6.9%)
Probably	1 (1.0%)	2 (2.0%)
Definite	0 (0%)	0 (0%)
**Causality to Challenge**		
Not-related	30 (29%)	24 (24%)
Possible	24 (23%)	24 (24%)
Probably	6 (5.8%)	8 (7.9%)
Definite	15 (15%)	11 (11%)
Not Applicable[2]	28 (27%)	34 (34%)
**AESI**		
No	97 (94%)	101 (100%)
Yes	6 (5.8%)	0 (0%)
**Ongoing**		
No	101 (98%)	98 (97%)
Yes	1 (1.0%)	3 (3.0%)
Upgraded to a SAE	1 (1.0%)	0 (0%)
**Days between AE start date and AE end date**	Mean: 26 (51), Med: 6 (0, 334)	Mean: 21 (53), Med: 6 (0, 384)
Unknown[3]	1	3

[1]*n (%); Mean: Mean (SD), Med: Median (Min, Max)*

[2]*For these participants, the stage of study for AE is 'Vaccination'. Therefore, the causality assessment to challenge will not appear to the clinical team (AEs occurred before the challenge, so it is not possible for the AE to be related to the challenge).*

[3]*Ongoing AEs.*

Table S6. Clinical and microbiological outcomes

**Outcomes in diagnosed participants**	**CVD group (n = 7)**	**Placebo group (n = 27)**
**Clinical outcomes**		
Time to diagnosis in days, median [IQR]	7.04 [5.97-12.47]	6.08 [5.26-8.39]
Fever ≥ 38°C related to paratyphoid diagnosis*, any duration, n(%)	2 (28.5%)	5 (19.5%)
Any symptom, n (%)	5 (71%)	22 (81%)
Any severe symptom, n (%)	2 (28%)	7 (25.9%)
Severe enteric fever, n (%)	0	0
Median maximum peak CRP, mg/L	16.8 [4.55-44.15]	18.5 [5.5 – 45.0]
**Microbiological outcomes**		
S. Paratyphi A bacteraemia, n (%)	7 (100%)	27 (100%)
Time to first positive blood culture in days, median [IQR]**	7.04 [5.97 – 12.47]	6.00 [5.00 – 8.39]
Duration of bacteraemia in days, median [range]	3.01 [0.92-6.04]	2.68 [0.95-6.06]
**Outcomes in all participants**	**CVD 1902 group (n = 34)**	**Placebo group (n = 36)**
Clinical diagnosis	0 (0%)	2 (5.5%)
Microbiological diagnosis	7 (20%)	25 (67%)
No of participants with at least 1 positive stool culture > 72 hours after challenge until D14/PD96	8 (23.5%)	18 (50%)
- Participants with 1 positive stool culture	- 4	- 9
- Participants with 2 positive stool cultures	- 1	- 5

- Participants with 3 positive stool cultures	- 1	- 2
- Participants with 4 positive stool cultures	- 0	- 2
- Participants with 5 or more positive stool cultures***	- 2	- 0
Time to first positive stool culture (excluding first 72 hours), in days, median [IQR]	10.85 [5.49 - 11.43]	6.7 [4.4 – 8.4]
Unique number of participants with $\geq$ 1 positive stool $\geq$ 1 week after completing antibiotics	3 (9%)	4 (11%)
- Single stool positive requiring re-treatment	- 3	- 2
- Further episode of stool positivity following re-treatment****	- 0	- 2
Bacteraemia relapse	0	3 (8%)
- 1 episode of bacteraemia relapse	- 0	- 2
- 2 episodes of bacteraemia relapse*****	- 0	- 1
Time from prior antibiotic completion to clinical symptoms of SPA relapse	NA	18 days

**One participant was diagnosed with paratyphoid on D7 and was afebrile throughout paratyphoid illness. They had a subsequent temperature > 38°C on D20 due to a diagnosed tonsillitis illness and therefore have not been included here.*

***Two participants were diagnosed on the basis of a persistent temperature > 38°C but also had a positive blood culture which was taken before their first temperature > 38°C, explaining why the numbers differ slightly from 'time to diagnosis'*

**** Two vaccine participants had > 5 positive stool cultures during the challenge period. One participant had 7 positive stools and the other had 10 positive stool cultures (see Figure S2).*

***** Two placebo participants had a further stool culture positive for S. Paratyphi A at least one week after completing their antibiotic re-treatment for initial stool positivity. Both participants required a further 14 day course of antibiotics and then completed 3 negative stool cultures following this.*

*********** *One participant had a second episode of relapse 18 days after completing 14 days of antibiotic treatment for first relapse episode. They completed a further 14-day course of antibiotics (3 days IV ceftriaxone followed by PO ciprofloxacin) for this second episode and were followed up the local Infectious Diseases team who gave a further course of antibiotic treatment (1 month ciprofloxacin 750mg BD). They had no further episodes throughout the study and completed 3 negative clearance stools by end of study.*

Table S7. Antibody titres to *S.* Paratyphi A specific antigens post vaccination

GMT = Geometric mean titres (in ELISA units). GMR = Geometric mean ratio between CVD 1902 and placebo at relevant timepoint.

	Study Timepoint	CVD 1902 GMT (95% CI)	Placebo GMT (95% CI)	GMR CVD 1902:Placebo (95% CI)	Fold rise from baseline, CVD 1902 (95% CI)	Fold rise from baseline, Placebo (95% CI)
**COPS IgG**	Baseline	126 (66-241)	163 (95-279)	0.77 (0.35 1.67)		
	14 days post first vaccine	314 (167-590)	160 (92-276)	1.97 (0.92-4.23)	2.50 (1.74 – 3.59)	0.98 (0.92 – 1.04)
	42 days post first vaccine (28 days post second vaccine)	292 (158-539)	166 (96 – 286)	1.76 (0.83-3.71)	2.32 (1.62- 3.34)	1.02 (0.96 – 1.08)
**COPS IgA**	Baseline	20 (16-26)	28 (20 - 40)	0.72 (0.53-0.99)		
	14 days post first vaccine	70 (49-99)	28 (20 - 39)	2.49 (1.67-3.71)	3.44 (2.44 - 4.85)	1.00 (0.94 – 1.05)
	42 days post first vaccine (28 days post second vaccine)	33 (24-45)	29 (21 – 41)	1.13 (0.80-1.62)	1.63 (1.15 – 2.29)	1.03 (0.98 -1.09)
**FliC IgG**	Baseline	39 (26 – 57)	44 (32- 62)	0.87 (0.56-1.35)		
	14 days post first vaccine	39 (27 – 57)	39 (28 – 56)	1.0 (0.65-1.52)	1.01 (0.94 – 1.09)	0.89 (0.73 – 1.07)
	42 days post first vaccine (28 days post second vaccine)	40 (28 – 58)	40 (28 – 56)	1.01 (0.66 – 1.53)	1.05 (0.97 – 1.13)	0.90 (0.75 – 1.09)
**FliC IgA**	Baseline	37 (27-51)	59 (43 – 82)	0.63 (0.44 – 0.90)		
	14 days post first vaccine	38 (28 – 52)	57 (42 – 79)	0.66 (0.47- 0.94)	1.01 (0.95 – 1.09)	0.90 (1.03 – 0.98)
	42 days post first vaccine (28 days post second vaccine)	38 (27 – 52)	58 (42- 79)	0.65 (0.47- 0.94)	1.01 (0.94 – 1.08)	0.98 (0.91 – 1.04)

Table S8. Antibody titres to *S.* Paratyphi A specific antigens post vaccination with two participants removed

During immunogenicity analysis it was noted that two participants had non-consistent results. Further investigation by site team revealed an error in serum tube labelling at the sample processing stage. Further investigation was done to confirm the error (both at site and with serum samples) and error was confirmed and therefore samples were correctly re-labelled for primary analysis. A sensitivity analysis was carried out removing both of these participants for completion – results shown here.

GMT = Geometric mean titres (in ELISA units/mL). GMR = Geometric mean ratio between CVD 1902 and placebo at relevant timepoint.

	**Study Timepoint**	**CVD 1902 GMT (95% CI)**	**Placebo GMT (95% CI)**	**GMR CVD 1902:Placebo**	**Fold rise from baseline (CVD 1902)**	**Fold rise from baseline (Placebo)**
**COPS IgG**	Baseline	141 (76-264)	148 (88-247)	0.95 (0.46 – 2.00)		
	14 days post first vaccine	348 (189 - 643)	145 (85-245)	2.41 (1.15 – 5.03)	2.47 (1.70 – 3.58)	0.98 (0.92 – 1.04)
	42 days post first vaccine (28 days post second vaccine)	324 (179-587)	152 (89-258)	2.13 (1.04 – 4.37)	2.30 (1.58 – 3.33)	1.03 (0.97 – 1.09)
**COPS IgA**	Baseline	21 (16-27)	28 (20-40)	0.74 (0.54 – 1.02)		
	14 days post first vaccine	67 (47-96)	28 (20-39)	2.41 (1.61 – 3.61)	3.23 (2.32 – 4.50)	0.99 (0.94 – 1.05)
	42 days post first vaccine (28 days post second vaccine)	34 (25-46)	29 (20-41)	1.15 (0.80- 1.66)	1.61 (1.16 – 2.24)	1.03 (0.97 – 1.09)
**FliC IgG**	Baseline	40 (27-59)	44 (31-63)	0.90 (0.58-1.42)		
	14 days post first vaccine	40 (28-59)	38 (27-55)	1.04 (0.68 – 1.61)	1.01 (0.94 – 1.09)	0.87 (0.72 – 1.06)
	42 days post first vaccine (28 days post second vaccine)	42 (29-61)	40 (28-56)	1.05 (0.68 – 1.61)	1.04 (0.97 – 1.13)	0.90 (0.74 – 1.09)
**FliC IgA**	Baseline	38 (28-53)	58 (42-81)	0.66 (0.46 – 0.95)		
	14 days post first vaccine	39 (28-54)	56 (41-78)	0.69 (0.48 – 0.99)	1.02 (0.94 – 1.09)	0.97 (0.90 – 1.03)
	42 days post first vaccine (28 days post second vaccine)	39 (28-54)	57 (42-79)	0.68 (0.47 – 0.98)	1.02 (0.95 – 1.10)	0.99 (0.92 – 1.05)

Table S9. Vaccine Efficacy by analysis population

	CVD 1902 group	Placebo group	RR (95% CI)	VE (95% CI)
	Attack Rate%	Attack Rate%	($AR_{CVD\ 1902}$/ $AR_{Placebo}$)	
**Intention-to-treat (ITT) population**	20.6% (7/34)	75% (27/36)	27.5% (13.8 - 54.5%)	72.5% (45.5 - 86.2%)
**Per-protocol (PP) population**	22.9% (8/35)	74.3% (26/35)	30.8% (16.2 - 58.3%)	69.2% (41.7 - 83.8%)

Table S10. Vaccine Efficacy by baseline anti-O IgG and interval between second vaccination and challenge

	CVD 1902 group	Placebo group	RR (95% CI)	VE (95% CI)
	Attack Rate%	Attack Rate%	($AR_{CVD\ 1902}$/ $AR_{Placebo}$)	
**Baseline *S.* Paratyphi O-antigen IgG antibody level**				
<= 148.5 EU/mL (Median baseline anti-O IgG)	17.6% (3/17)	66.7% (12/18)	26.5% (9 - 77.8%)	73.5% (22.2 - 91%)
> 148.5 EU/mL (Median baseline anti-O IgG)	23.5% (4/17)	83.3% (15/18)	28.2% (11.7 - 68.2%)	71.8% (31.8 - 88.3%)
**Interval between second vaccination and challenge**				
<= 28 days (Median interval)	25% (5/20)	81.2% (13/16)	30.8% (13.9 - 68.1%)	69.2% (31.9 - 86.1%)
> 28 days (Median interval)	14.3% (2/14)	70% (14/20)	20.4% (5.5 - 76%)	79.6% (24 - 94.5%)
**Baseline S.Paratyphi O-antigen IgG antibody level**				
<= 802.2 EU/mL (GMT+1SD)	24.1% (7/29)	75% (21/28)	32.2% (16.3 - 63.5%)	67.8% (36.5 - 83.7%)
> 802.2 EU/mL (GMT+1SD)*	0% (0/5)	75% (6/8)	-	-

* RR and VE cannot be calculated as there were no events in the CVD 1902 group.

Table S11. Representativeness of study participants

Category	Details
Disease under investigation	*Salmonella enterica* Paratyphi A, cause of paratyphoid/enteric fever
Special considerations related to:	
Sex and gender	*S.* Paratyphi A affects both sexes equally (1)
Age	Incidence rates are highest amongst children, peaking in the school-age (5-9 year) age group, with 55% of cases occurring in children younger than 15 years (2).
Race or ethnic group	All ethnic groups are vulnerable to *S.* Paratyphi A but the disease is most prevalent in South Asia, therefore South Asians are disproportionately affected (2).
Geography	The highest incidence of *S.* Paratyphi A is in South Asia. In 2017, over 80% of global paratyphoid cases were in South Asia. Western sub-Saharan Africa, East Asia and Southeast Asia also had high levels of cases in 2017 (2).
Other considerations	As a faeco-orally transmitted disease, there is an association between poor hygiene and untreated water and risk of enteric fever (3, 4). There is evidence of a seasonal pattern of enteric fever, with seasonal variability more pronounced further from the equator. Increased rainfall also increases the risk of enteric fever in endemic areas (5).<br>*S.* Paratyphi A is seen in non-endemic countries, almost exclusively related to travel. For example in 2023, 204 cases of SPA were seen in the UK, representing 37% of all enteric fever cases in the UK (6).
Overall representativeness of this study	The participants in this study were enteric fever-naïve healthy UK adults between 18-55 (median age of 31.5 years). Birth sex and gender were self-reported with a female: male ratio of 46:54% in this study. The majority of participants in this study were of white ethnicity (84%). This population therefore differs from that of the population most

	**affected by SPA. It is not feasible to conduct an SPA CHIM in young children, however, UK adults may be similarly naïve to paratyphoid infection as young children in endemic areas.**

References

1. Evaluation IfHMa. Paratyphoid fever - Level 4 cause [Available from: https://www.healthdata.org/research-analysis/diseases-injuries-risks/factsheets/2021-paratyphoid-fever-level-4-disease.

2. Stanaway JD, Reiner RC, Blacker BF, Goldberg EM, Khalil IA, Troeger CE, et al. The global burden of typhoid and paratyphoid fevers: a systematic analysis for the Global Burden of Disease Study 2017. The Lancet Infectious Diseases. 2019;19(4):369-81.

3. Brockett S, Wolfe MK, Hamot A, Appiah GD, Mintz ED, Lantagne D. Associations among Water, Sanitation, and Hygiene, and Food Exposures and Typhoid Fever in Case-Control Studies: A Systematic Review and Meta-Analysis. Am J Trop Med Hyg. 2020;103(3):1020-31.

4. Meiring JE, Khanam F, Basnyat B, Charles RC, Crump JA, Debellut F, et al. Typhoid fever. Nat Rev Dis Primers. 2023;9(1):71.

5. Saad NJ, Lynch VD, Antillon M, Yang C, Crump JA, Pitzer VE. Seasonal dynamics of typhoid and paratyphoid fever. Sci Rep. 2018;8(1):6870.

6. Agency UHS. Enteric fever (typhoid and paratyphoid) England, Wales and Northern Ireland 2023: UK Health Security Agency 2024 [cited 2025 28/04/2025]. Available from: https://www.gov.uk/government/publications/typhoid-and-paratyphoid-laboratory-confirmed-cases-in-england-wales-and-northern-ireland/enteric-fever-typhoid-and-paratyphoid-england-wales-and-northern-ireland-2023.
